# Supplementary material for: Age, gender and socioeconomic patterns of awareness and usage of e-cigarettes across selected WHO region countries: evidence from the Global Adult Tobacco Survey
Source: BMJ Open. 2023 Jan 19;13(1):e070419. doi: 10.1136/bmjopen-2022-070419 (PMC9853219; doi:10.1136/bmjopen-2022-070419)
Supplement: Supplementary data [file bmjopen-2022-070419supp001.pdf]

Title: Age, gender and socio-economic patterns of awareness and usage of e-cigarettes across selected WHO region countries: evidence from the Global Adult Tobacco Survey

Table S1: Age-sex standardized prevalence of current e-cigarette use, GATS, 2011-2017

WHO Region	Country	Survey Year	Prevalence (%)
African Region	Ethiopia	2016	1.49
	Senegal	2017	0.64
Eastern Mediterranean	Qatar	2013	1.97
European Region	Greece	2013	1.64
	Kazakhstan	2014	2.87
	Russian Federation	2016	3.74
	Ukraine	2017	3.05
Region of the Americas	Costa Rica	2015	2.48
	Mexico	2015	1.59
South-east Asia Region	Indonesia	2011	1.07
	India	2017	1.66
Western Pacific Region	Malaysia	2011	1.93
	Philippines	2015	1.97
	Vietnam	2015	0.79
