# Supplementary material for: Age, gender and socioeconomic patterns of awareness and usage of e-cigarettes across selected WHO region countries: evidence from the Global Adult Tobacco Survey
Source: BMJ Open. 2023 Jan 19;13(1):e070419. doi: 10.1136/bmjopen-2022-070419 (PMC9853219; doi:10.1136/bmjopen-2022-070419)
Supplement: Author's manuscript [file bmjopen-2022-070419.draft_revisions.pdf]

BMJ Open is committed to open peer review. As part of this commitment we make the peer review history of every article we publish publicly available.

When an article is published we post the peer reviewers' comments and the authors' responses online. We also post the versions of the paper that were used during peer review. These are the versions that the peer review comments apply to.

The versions of the paper that follow are the versions that were submitted during the peer review process. They are not the versions of record or the final published versions. They should not be cited or distributed as the published version of this manuscript.

BMJ Open is an open access journal and the full, final, typeset and author-corrected version of record of the manuscript is available on our site with no access controls, subscription charges or pay-per-view fees (http://bmjopen.bmj.com).

If you have any questions on BMJ Open's open peer review process please email info.bmjopen@bmj.com

BMJ Open

# Awareness and determinants of e-cigarette use across selected WHO region countries: Evidence from the Global Adult Tobacco Survey (GATS)

| | |
|---|---|
| Journal: | *BMJ Open* |
| Manuscript ID | bmjopen-2022-070419 |
| Article Type: | Original research |
| Date Submitted by the Author: | 26-Nov-2022 |
| Complete List of Authors: | Kundu, Sampurna; Jawaharlal Nehru University, Centre of Social Medicine and Community Health<br>Shaw, Subhojit; International Institute for Population Sciences, Department of Population and Development<br>Khan, Junaid; International Institute for Population Sciences, Department of Population and Development<br>Chattopadhyay, Aparajita; International Institute for Population Sciences, Department of Population and Development<br>Baptista, Emerson; El Colegio de Mexico AC<br>Paswan, Balram; International Institute for Population Sciences |
| Keywords: | PUBLIC HEALTH, SOCIAL MEDICINE, Health policy < HEALTH SERVICES ADMINISTRATION & MANAGEMENT |
| | |

SCHOLARONE™
Manuscripts

*I, the Submitting Author has the right to grant and does grant on behalf of all authors of the Work (as defined in the below author licence), an exclusive licence and/or a non-exclusive licence for contributions from authors who are: i) UK Crown employees; ii) where BMJ has agreed a CC-BY licence shall apply, and/or iii) in accordance with the terms applicable for US Federal Government officers or employees acting as part of their official duties; on a worldwide, perpetual, irrevocable, royalty-free basis to BMJ Publishing Group Ltd ("BMJ") its licensees and where the relevant Journal is co-owned by BMJ to the co-owners of the Journal, to publish the Work in this journal and any other BMJ products and to exploit all rights, as set out in our licence.*

*The Submitting Author accepts and understands that any supply made under these terms is made by BMJ to the Submitting Author unless you are acting as an employee on behalf of your employer or a postgraduate student of an affiliated institution which is paying any applicable article publishing charge ("APC") for Open Access articles. Where the Submitting Author wishes to make the Work available on an Open Access basis (and intends to pay the relevant APC), the terms of reuse of such Open Access shall be governed by a Creative Commons licence – details of these licences and which Creative Commons licence will apply to this Work are set out in our licence referred to above.*

*Other than as permitted in any relevant BMJ Author's Self Archiving Policies, I confirm this Work has not been accepted for publication elsewhere, is not being considered for publication elsewhere and does not duplicate material already published. I confirm all authors consent to publication of this Work and authorise the granting of this licence.*

For peer review only

# Awareness and determinants of e-cigarette use across selected WHO region countries: Evidence from the Global Adult Tobacco Survey (GATS)

Sampurna Kundu[1], Subhojit Shaw[2], Junaid Khan[2], Aparajita Chattopadhyay[2], Emerson Augusto Baptista[3,*], Balram Paswan[2]

[1] Jawaharlal Nehru University, New Delhi, Delhi 110067, India.

[2] International Institute for Population Sciences, Deonar, Mumbai 400088, India.

[3] El Colegio de México, CEDUA, Mexico City 14110, México.

* Correspondence to Prof. Aparajita Chattopadhyay; aparajita@iipsindia.ac.in

**Word count:** 4464

## Abstract

**Objectives** The purpose of this study is to describe awareness and prevalence of e-cigarette use by demographic and socio-economic characteristics of the study population (≥15 years) in selected 14 GATS countries.

**Design** Cross-sectional.

**Setting** 14 countries.

**Participants** Country-specific surveyed population (≥15 years) constitute the study sample. Individuals were selected using multi-stage cluster sampling design.

**Primary and secondary outcome measures** We selected 14 countries from six different WHO regions where Global Adult Tobacco Survey (GATS) was conducted in different years during 2011-2017. Using bivariate cross-tabulation, we estimated the prevalence of e-cigarette use by socio-economic and demographic characteristics. In addition, country specific multivariable logistic regression was estimated to examine the pattern and determinants of e-cigarette use in the respective countries.

**Results** Among the selected GATS countries, awareness of e-cigarette was highest in Greece (88%), whereas it was observed to be lowest in India (3%). On the other hand, the prevalence of e-cigarette use was highest in Russia (4%) and lowest in India (0.7%). Across all countries the prevalence of e-cigarette use is higher among males except Greece. A few European and American region countries have reported high use of e-cigarette among younger age group (15-24). In general, the logistic estimation showed that those who are of better socio-economic condition (rich, higher educated, resides in urban areas) are more likely to use e-cigarette than their counterparts. When adjusted for all other factors, the age-associated estimates of adjusted odds ratio informed no consistent pattern of likelihood across the selected countries.

**Conclusions** This study shows that the urban population, males, young people aged 15-24, people with higher wealth index scores and the educated population have a better knowledge of e-cigarette across all the countries. As e-cigarette use is much more prevalent among young adults, their behaviors also need to be closely monitored at different levels.

**Keywords**: E-cigarette; GATS; Global Health; Public Policy; Tobacco Smokers.



For peer review only - http://bmjopen.bmj.com/site/about/guidelines.xhtml

**Strengths and limitations of this study**

- This study provides an important baseline e-cigarette prevalence data for multiple countries.
- This cross-country study explores the factors affecting adolescent and adult (≥ 15 years) behavior of e-cigarettes usage in 14 selected GATS countries.
- Among the current tobacco smokers, the likelihood of e-cigarette use is higher across all the study countries.
- A limitation to this study is that the estimates of e-cigarette awareness and its use are based on self-reported information.
- As GATS data collections continue, the prevalence of e-cigarette use in each country can continue to be monitored and thus can be used to evaluate the existing policies over time.

For peer review only



For peer review only - http://bmjopen.bmj.com/site/about/guidelines.xhtml

## Introduction

The use of e-cigarettes has become common among youths and there are concerns that widespread use may lead to future epidemic[1–3]. Subsequently, the popularity and use of e-cigarettes have increased over the years[4]. E-cigarettes and other battery-powered vaporizers were first launched in China, 2003 and later on entered the US market in 2007[5]. Surprisingly, during 2016, an estimated 54.6% of the e-cigarette users were also conventional cigarette smokers among US adults[6]. The use of e-cigarettes have evolved as a means of reducing the harmful effects of smoking while still delivering nicotine. E-cigarettes vaporize a liquid that contains nicotine for inhalation without the need of burning leaf materials. Despite of its widespread adoption and high public exposure, there remains a disparity in the diffusion of e-cigarette awareness among different socio-demographic groups[4].

There is no consensus in the previous literatures on the long-term effect or benefits of quitting smoking with e-cigarettes[7]. A few studies suggest that e-cigarettes can serve as a gateway for subsequent tobacco smoking[8–11]. According to a cross-sectional study in the USA, e-cigarette use is low among former smokers than the current adult smokers [12]. A study conducted by the United States Food and Drug Administration (FDA) revealed that e-cigarettes contain carcinogens, nitrosamines, diethylene glycol and other chemicals [13]. These chemicals have adverse effects on children, adolescents, and pregnant mothers, contributing to cardiovascular diseases [14–16]. E-cigarettes are also available in varied flavors, making them more attractive and appealing to the youths [17]. For older adult smokers, e-cigarettes possess a beneficial health transition; however, amateur young adults who use e-cigarettes have a potential health risk. While nicotine itself is not a carcinogen, malignant diseases and neurodegeneration are suspected to result from nicotine [14]. A legitimate concern among the children is that if children (non-smokers) develops a nicotine addiction, they may start smoking cigarettes [18]. A recent study has suggested that young adults who use e-cigarettes had higher odds of conventional smoking initiation[19]. Furthermore, a report published by the National Academies of Sciences, Engineering, and Medicine (2018) had found evidence of an increase in the risk of tobacco smoking due to e-cigarette use among young adults [15]. Hence, this poses a major public health challenge that requires strict regulation to access e-cigarettes.

A study conducted in the USA had shown that e-cigarette usage rates increased from 1.5% to 20.8% among youths during 2011-2018 [20]. The use of e-cigarettes among youth adolescents has raised concerns about a new generation's lifelong addiction. Some previous literatures showed an increase in the use of e-cigarettes among the youth, especially those in the higher economic sections [21,22]. The diffusion and innovation theory by Everett Rogers, proposed in 1962, suggested that innovations are first appreciated by the upper class, followed by others [23,24]. E-cigarette use mimics low nicotine, reduces tar exposure, and is more aesthetically appealing than other forms of smoking, making it more attractive alternative with a higher rate of adaptation [25]. Therefore, an understanding of e-cigarette usage and its proximate determinants factors across different nations is critical. A handful of studies found that the likelihood of e-cigarette use was higher in the event of exposure to advertisements/promotions [26–28]. Since 2014, the Institute for Global Tobacco Control (IGTC), under Johns Hopkins Bloomberg School of Public Health, has been working globally across 120 nations to measure tobacco



For peer review only - http://bmjopen.bmj.com/site/about/guidelines.xhtml

advertisement, promotion and evaluate the e-cigarette policy. In 2015, the WHO and World Bank Group called on WHO member countries to increase tobacco and related product taxes to prevent youths from initiating tobacco use [29]. In subsequent years, through numerous policies and programs, the WHO offered to help fight the tobacco epidemic [30]. In collaboration, WHO member countries have developed mechanisms for tobacco cessation support. At the same time, the amount and quality of scientific evidence have not been sufficient to determine whether e-cigarettes may help most smokers to quit or prevent tobacco smoking [31]. According to the WHO, member countries should regulate e-cigarettes to avoid promotion and intake by non-smokers, pregnant women, and youths [32].

A systematic review of literatures by Hartwell et al., 2017, has shown that the usage of e-cigarettes is higher among young adults, in higher socio-economic classes, and among individuals with a higher level of education [4]. The use of e-cigarettes is on the rise and a significant amount of research has been undertaken from a small selection of high-income countries [1,2,8,12,33,34]. In general, the use of e-cigarette is increasing among young adults as well as among the general adult population across many countries. GATS surveys in its member countries lately introduced a set of questions to collect the self-reported information on e-cigarette use and its awareness. Given the list of GATS survey countries, there exists no holistic research exploring the socio-economic and demographic determinants of e-cigarette awareness and its use. In this context, the present cross-country study explores the factors affecting adolescent and adult (population over 15 years) behavior of e-cigarettes usage, employing the most recent round of the Global Adult Tobacco Survey (GATS) datasets. From a socio-political perspective, this study is a timely contribution to identify the sub-population at higher risk of using e-cigarettes.

**Data**

Global Adult Tobacco Survey (GATS), an integral part of the Global Tobacco Surveillance System (GTSS), is a nationally representative household survey started in 2008. The survey collects specific information on tobacco use and tracks key tobacco control indicators among non-institutionalized adults 15 years of age or older in GATS countries. GATS uses country-specific stratified multi-stage cluster sampling designs in which probability proportional to size (PPS) random selection methods are used to successively choose clusters in one or more steps to secure ample coverage of the target population. The sample of households is chosen in two or more stages, with sampling units in the first or second stage being well-defined geopolitical areas within the country. These areas are then randomly selected from a complete list of enumeration areas having no more than 250 households. At the final stage, households were surveyed from the randomly selected areas. From the selected household, one individual aged 15 years or older was randomly chosen to participate in the survey. The collection of information was carried out using electronic handheld devices. The overall response rate ranged from 64.4% to 98.5% across the selected counties in the study. For the first time since 2011, GATS introduced the questions on e-cigarette awareness and the current use of e-cigarettes [35]. Table 1 shows the GATS datasets being included in the study.



For peer review only - http://bmjopen.bmj.com/site/about/guidelines.xhtml

**Table 1**. Description of the GATS dataset included in the study

| WHO Regions | Country | Survey Year | Households Surveyed | Individuals Interviewed | Response Rate (%) |
|---|---|---|---|---|---|
| African Region | Ethiopia | 2016 | 10,875 | 10,150 | 93.4 |
| | Senegal | 2017 | 4,514 | 4,347 | 97.0 |
| Eastern Mediterranean | Qatar | 2013 | 8,571 | 8,398 | 98.5 |
| European Region | Greece | 2013 | 6,600 | 4,359 | 69.6 |
| | Kazakhstan | 2014 | 4,611 | 4,425 | 96.7 |
| | Russian Federation | 2016 | 11,764 | 11,458 | 98.2 |
| | Ukraine | 2017 | 14,800 | 8,298 | 64.4 |
| Region of the Americas | Costa Rica | 2015 | 9,680 | 8,607 | 89.2 |
| | Mexico | 2015 | 17,765 | 14,664 | 82.7 |
| South-east Asia Region | Indonesia | 2011 | 8,994 | 8,305 | 94.3 |
| | India | 2017 | 84,047 | 74,037 | 92.9 |
| Western pacific Region | Malaysia | 2011 | 5,112 | 4,250 | 85.3 |
| | Philippines | 2015 | 13,963 | 11,644 | 88.4 |
| | Vietnam | 2015 | 9,514 | 8,996 | 95.8 |

***Note:*** Compiled by authors

The surveys across the countries tracked down the awareness of e-cigarettes at different survey time points. In the survey questionnaire, e-cigarettes have been described as "Electronic cigarettes include any product that uses batteries or other methods to produce a vapor which contains nicotine. The description also includes various other names such as e-cigarette, vape-pen, e-shisha, e-pipes". The respondents were then asked "Have you ever heard of e-cigarettes?" Those who responded 'yes' were considered to be aware of e-cigarettes. Those individuals who were aware of e-cigarettes were further asked to respond if he/she had currently used e-cigarettes on a daily basis, less than daily, or not at all. Those who responded as 'daily' or 'less than daily' were considered current users of e-cigarettes. In this study, we selected those fourteen countries that collected the specific information on e-cigarette use and its awareness in their most recent round of the GATS surveys (Table 1) [36–38].

### *Outcome variable*

The key outcome variable of this study was the respondent's current e-cigarette use status and was defined as a dichotomous (yes/no) variable. The variable was defined as '1' if the respondent answered as currently using e-cigarettes on 'daily' or 'less than daily' basis and '0' otherwise.

### *Independent variables*

Among the independent variables, the study utilized various socio-economic and demographic characteristics of the study population such as gender, age, residence, wealth index, education and occupation. According to the sample distribution, age was recoded into four age-groups: 15-24, 25-44, 45-64, and above 64. Education was categorized as: no formal education/less than primary, completed primary/less than secondary, completed secondary or completed high school, and completed college or university or above. The 'occupation' variable included two categories, 'employed' and 'non-employed/unemployed'.



For peer review only - http://bmjopen.bmj.com/site/about/guidelines.xhtml

Wealth quintile variable has been computed in this study to measure the economic status of the respondents, which is commonly used in the cross sectional surveys [39]. Authors have followed the standard DHS framework to compute the wealth variable using principal component analysis (PCA) [40]. While this is a common computation method of creating wealth index variable where households are given scores based on the number and kinds of consumer goods it own, ranging from electricity connectivity, flash toilet, fixed telephone, cell telephone, television, radio, car, refrigerator, scooter or motor cycle, washing machine, computer or laptop, internet connection, air conditioner and electric fan. Then each individual from the same household are ranked by the scores and the distribution was equally divided into five quintile categories. of the bottom 20% of the population is identified as poorest, next 20% as poorer, and likewise the top 20% is identified as the richest. In the present study we have further recoded the wealth quintile variable into three categories, such as – poor, middle and rich.

The current tobacco use status was assessed by the question ‘Do you currently smoke tobacco on a daily basis, less than daily, or not at all?’. People who responded ‘daily’ or ‘less than daily’ were considered as current tobacco smokers (coded 1) and ‘not at all’ were considered non-smokers (coded 0). The tobacco products used included manufactured cigarettes, cigars, pipes, hand-rolled cigarettes, kreteks, and water pipes.

The study used two types of noticing to advertisements- in stores and on the internet. The store advertisement noticing was measured by the question ‘In the past 30 days, have you noticed any advertisements or signs promoting cigarettes in stores where cigarettes are sold?’, and, similarly, internet advertisement noticing was measured by the question ‘In the past 30 days, have you noticed any advertisements or signs promoting cigarettes on the internet?’ Respondents who said ‘Yes’ were the individuals to notice the advertisements (coded 1), and coded ‘No’ as ‘0’ otherwise.

**Statistical Analysis**

For each selected country, we calculated the prevalence of current use of e-cigarettes, as well as the percentage of awareness about e-cigarettes. Further, we used bivariate analysis in the form of chi-square test of independence and cross-tabulations as well. To examine the socio-economic and demographic determinants of e-cigarette use in the population of the respective countries, we employed country specific multivariable logistic regression. The general equation of the logistic equation is as follows-

$$logit\left(\frac{p_i}{1-p_i}\right) = \alpha + \beta_1(gender_i) + \beta_2(age_i) + \beta_3(residence_i) + \beta_4(education_i) + \beta_5(occupation_i) + \beta_6(wealth\ index_i) + \beta_7(CST_i) + \beta_8(Ad_{stores_i}) + \beta_9(Ad_{internet_i}) + \varepsilon_i \quad (29)$$

Here $\alpha$ is the intercept, $\beta_j s$ are the coefficients and $\varepsilon_i$ is the error component. CST refers to current smoking of tobacco, ‘Ad_stores’ refers to the variable of noticing advertisements in stores and similarly ‘Ad_internet’ refers to noticing advertisements on internet.



For peer review only - http://bmjopen.bmj.com/site/about/guidelines.xhtml

Once the logistic model was fitted for a particular country, we estimated the predicted prevalence from the model fit which gave the average prevalence of e-cigarette use after adjusting for all other socio-economic and demographic factors for that country. The 'logit' package in STATA provides different features for post-estimation. There are different post-estimation commands available from the same package and we used the 'predict' command which by default estimates the probability of the positive outcome (Y=1). This 'predict' command generates a new variable to store the estimated probability for each of the study individual/entity. Thus, for each of the country specific fitted logistic model, we estimated the average probability of the positive outcome, which is actually the average probability of using e-cigarette in the study population and hence the average predicted prevalence of e-cigarette use in a particular country. Sample weights ('gatsweight' in the survey dataset) have been used throughout the analysis. STATA version 14.1 was used to analyse the datasets.

**Patient and Public Involvement**

No patient involved

**Results**

***Awareness and current usage of e-cigarettes***

Figure 1 shows awareness and current usage of e-cigarettes across selected countries. The percentage of people with awareness of e-cigarettes is highest in Greece (88.46%), followed by Russia (79.84%) and Kazakhstan (53.69%), that is, countries located in the European region. At the other extreme, India is the country with lowest percentage of people with awareness of e-cigarettes (3.03%), followed by Ethiopia (3.99%), and Indonesia (10.95%). Hence, overall, the countries belonging to the European region shows a maximum level of awareness in comparison to countries of other WHO regions.

**Figure 1**. The prevalence of e-cigarettes usage and awareness across selected GATS countries

[Fig 1 about here]

*Compiled by authors, Source: https://nccd.cdc.gov/GTSSDataSurveyResources/Ancillary/DataReports.aspx?CAID=2*

Table 2 also shows that awareness of e-cigarettes is higher among males and younger people. In addition, e-cigarette awareness is higher among those with more education (completed high school and higher), higher wealth index scores, noticing advertisement, living in urban areas, employed, and those who are currently smoking tobacco. In summary, the chi-square test (Table 2) shows that there is a significant association between awareness of e-cigarettes and gender, age, place of residence, educational level, occupation, wealth quintile, current use of tobacco smoking and noticing of advertisement for almost all GATS countries studied. Exceptions are observed for Qatar, in Eastern Mediterranean, where there is no significant association of awareness with noticing advertising in stores.



For peer review only - http://bmjopen.bmj.com/site/about/guidelines.xhtml

Regarding the prevalence of current use of e-cigarettes, it is found to be generally low in majority of the selected GATS countries. The highest percentage of current usage of e-cigarettes is observed in Russia (4.39%), followed by Costa Rica (2.69%). On the other hand, countries like India (0.66%) and Senegal (0.73%) in the South-East Asia and African regions respectively shows lowest current use(Table 3). For Senegal (African region) and Vietnam (West Pacific region), although the prevalence is higher among men, there is no significant association observed between gender and current use of e-cigarettes . Few countries in European and American regions reflect high usage among the younger age groups (15-24 age): Russia (10.5%), Ukraine (7.6%), and Costa Rica (4.87%). In the American region, Costa Rica has the higher usage overall, but it is significantly associated with gender, age, residence and current tobacco smoking only. In addition, few countries show high usage among older age groups (64 age and above): Philippines (2.42%), India (1.51%), and Greece (1.25%), which are in the Western Pacific, South-east Asia and European region, respectively (Table 3). In terms of the age groups, there are significant differences observed in current e-cigarette use across the countries of Costa Rica, Greece, Mexico, and Ukraine. At the same time, except Greece and Russia (European region), none of the countries had any significant association of current use of e-cigarettes with educational level, although people with higher levels of education make more use of e-cigarettes (completed college or university, Greece: 4.5% and Russia: 4.7%). In all countries, the prevalence of current e-cigarette use is observed to be higher among those who smoke tobacco than the non-smokers. It is also found that current usage of smoking tobacco has a significant association with current use of e-cigarettes in the population across all the countries, except for Senegal (users: 0.9%, non-users: 0.72%) (Table 3).

For peer review only

**Table 2:** Country specific bi-variate estimation on awareness of e-cigarette use, GATS, 2011-2017

| Predictors | African Region | | | | Eastern Mediterranean | | European Region | | | | | | | | Regions of the Americas | | | | South-East Asia Region | | | | Western Pacific Region | | | | | |
|---|---|---|---|---|---|---|---|---|---|---|---|---|---|---|---|---|---|---|---|---|---|---|---|---|---|---|---|---|
| | Ethiopia | | Senegal | | Qatar | | Greece | | Kazakhstan | | Russian Federation | | Ukraine | | Costa Rica | | Mexico | | Indonesia | | India | | Malaysia | | Philippines | | Vietnam | |
| | % | $\chi^2$ | % | $\chi^2$ | % | $\chi^2$ | % | $\chi^2$ | % | $\chi^2$ | % | $\chi^2$ | % | $\chi^2$ | % | $\chi^2$ | % | $\chi^2$ | % | $\chi^2$ | % | $\chi^2$ | % | $\chi^2$ | % | $\chi^2$ | % | $\chi^2$ |
| **Gender** | | | | | | | | | | | | | | | | | | | | | | | | | | | | |
| Male | 5.23 | 58.90*** | 18.70 | 74.21*** | 62.80 | 835.79*** | 93.43 | 129.39*** | 64.06 | 174.80*** | 86.28 | 315.84*** | 58.04 | 216.63*** | 51.98 | 58.53*** | 40.93 | 193.02*** | 16.83 | 307.30*** | 4.14 | 313.67*** | 29.00 | 181.44*** | 36.36 | 122.65*** | 25.53 | 352.65*** |
| Female | 2.74 | | 9.73 | | 33.81 | | 83.67 | | 44.36 | | 74.52 | | 44.96 | | 42.94 | | 30.15 | | 5.09 | | 1.87 | | 12.43 | | 27.10 | | 12.01 | |
| **Age** | | | | | | | | | | | | | | | | | | | | | | | | | | | | |
| 15-24 | 2.96 | 19.69*** | 12.56 | 33.56*** | 48.78 | 78.31*** | 93.73 | 803.79*** | 59.56 | 350.55*** | 91.45 | 2400.00*** | 63.09 | 921.62*** | 51.93 | 207.56*** | 43.91 | 379.98*** | 14.24 | 141.08*** | 3.75 | 165.95*** | 24.11 | 139.43*** | 35.75 | 250.85*** | 20.97 | 143.86*** |
| 25-44 | 5.24 | | 16.30 | | 50.17 | | 95.22 | | 62.24 | | 92.26 | | 61.72 | | 51.72 | | 37.74 | | 12.53 | | 3.54 | | 26.17 | | 35.30 | | 21.64 | |
| 45-64 | 3.64 | | 14.81 | | 49.52 | | 35.21 | | 47.18 | | 80.17 | | 51.20 | | 43.56 | | 30.51 | | 7.89 | | 2.12 | | 13.06 | | 26.61 | | 15.64 | |
| above 64 | 3.96 | | 5.09 | | 25.83 | | 62.17 | | 19.54 | | 44.11 | | 24.30 | | 28.91 | | 15.21 | | 1.72 | | 0.91 | | 7.57 | | 12.54 | | 7.90 | |
| **Residence** | | | | | | | | | | | | | | | | | | | | | | | | | | | | |
| Urban | 8.27 | 176.61*** | 24.22 | 362.19*** | NA | NA | 90.43 | 73.74*** | 63.42 | 225.18*** | 81.98 | 211.83*** | 55.89 | 247.33*** | 51.80 | 248.05*** | 41.17 | 1100.00*** | 15.34 | 181.09*** | 5.12 | 683.54*** | 23.05 | 38.55*** | 43.83 | 576.93*** | 27.51 | 195.41*** |
| Rural | 2.62 | | 3.97 | | NA | | 83.25 | | 40.97 | | 73.46 | | 39.58 | | 35.21 | | 13.71 | | 6.53 | | 1.93 | | 15.55 | | 21.11 | | 14.00 | |
| **Education** | | | | | | | | | | | | | | | | | | | | | | | | | | | | |
| No formal education | 2.42 | 348.46*** | 2.97 | 782.24*** | 15.95 | 328.34*** | 49.85 | 809.60*** | 36.84 | 221.97*** | 33.44 | 1100.00*** | 49.25 | 503.06*** | 28.48 | 462.22*** | 9.40 | 1500.00*** | 1.40 | 575.35*** | 0.70 | 2300.00*** | 6.56 | 292.71*** | 13.16 | 840.25*** | 4.11 | 725.71*** |
| Completed primary | 3.26 | | 26.34 | | 39.68 | | 74.55 | | 38.70 | | 56.29 | | 15.07 | | 37.84 | | 21.22 | | 5.74 | | 2.13 | | 13.21 | | 21.25 | | 9.57 | |
| Completed secondary | 5.07 | | 35.98 | | 48.53 | | 94.43 | | 56.43 | | 79.08 | | 49.57 | | 50.04 | | 42.39 | | 16.08 | | 4.27 | | 23.27 | | 23.10 | | 20.76 | |
| Completed college/university | 13.99 | | 59.68 | | 56.52 | | 96.50 | | 64.02 | | 89.33 | | 61.28 | | 68.05 | | 59.20 | | 29.41 | | 10.07 | | 43.28 | | 50.30 | | 39.90 | |
| **Occupation** | | | | | | | | | | | | | | | | | | | | | | | | | | | | |
| Non employed | 2.35 | 25.56*** | 12.07 | 25.57*** | 36.00 | 604.03*** | 82.72 | 291.99*** | 41.10 | 309.02*** | 65.19 | 1400.00*** | 41.29 | 538.77*** | 40.94 | 184.19*** | 30.38 | 247.09*** | 8.89 | 28.85*** | 2.66 | 47.21*** | 15.58 | 105.77*** | 28.08 | 38.85*** | 16.93 | 16.32*** |
| Employed | 5.59 | | 16.23 | | 59.14 | | 96.58 | | 65.11 | | 90.47 | | 61.69 | | 54.73 | | 40.02 | | 12.16 | | 3.37 | | 25.19 | | 34.06 | | 19.15 | |
| **Wealth Index** | | | | | | | | | | | | | | | | | | | | | | | | | | | | |
| Rich | 7.76 | 269.44*** | 25.74 | 248.38*** | NA | NA | 93.57 | 398.44*** | 66.06 | 305.87*** | 86.09 | 614.97*** | 61.24 | 731.61*** | 54.79 | 271.09*** | 48.67 | 1500.00*** | 18.49 | 432.58*** | 5.86 | 1400.00*** | 27.46 | 185.19*** | 44.73 | 847.92*** | 31.08 | 615.73*** |
| Middle | 2.66 | | 9.47 | | NA | | NA | | 50.71 | | 75.80 | | 46.04 | | 42.99 | | 25.96 | | 8.07 | | 1.79 | | 19.35 | | 25.94 | | 16.54 | |
| Poor | 2.85 | | 2.60 | | NA | | 74.90 | | 37.72 | | 69.26 | | 32.09 | | 37.77 | | 14.80 | | 3.15 | | 1.28 | | 9.02 | | 15.97 | | 8.11 | |
| **Currently smoke tobacco** | | | | | | | | | | | | | | | | | | | | | | | | | | | | |
| No | 3.75 | 70.39*** | 13.66 | 7.17*** | 44.41 | 529.69*** | 83.93 | 219.58*** | 48.35 | 184.62*** | 74.97 | 493.53*** | 46.72 | 263.43*** | 45.00 | 194.59*** | 31.46 | 592.80*** | 7.75 | 163.63*** | 2.88 | 33.89*** | 17.60 | 101.71*** | 29.43 | 63.30*** | 16.91 | 67.99*** |
| Yes | 10.10 | | 21.49 | | 82.68 | | 95.79 | | 72.16 | | 91.07 | | 65.02 | | 72.79 | | 55.03 | | 16.94 | | 4.24 | | 32.11 | | 39.52 | | 24.25 | |
| **Advertisements in stores** | | | | | | | | | | | | | | | | | | | | | | | | | | | | |
| No | 3.95 | 19.15*** | 12.44 | 64.13*** | 48.91 | 0.59 | 85.38 | 120.96*** | 53.75 | 0.01*** | 79.33 | 37.97*** | 50.01 | 39.73*** | 46.47 | 23.91*** | 33.08 | 109.49*** | 7.93 | 124.73*** | 2.60 | 327.46*** | 19.55 | 22.53*** | 28.85 | 66.40*** | 17.15 | 99.34*** |
| Yes | 15.38 | | 31.35 | | 49.93 | | 96.26 | | 53.27 | | 89.22 | | 56.49 | | 57.00 | | 39.22 | | 14.54 | | 7.17 | | 27.00 | | 35.94 | | 33.49 | |
| **Advertisements on internet** | | | | | | | | | | | | | | | | | | | | | | | | | | | | |
| No | 3.96 | 11.31*** | 13.62 | 86.62*** | 48.20 | 6.90*** | 88.13 | 16.81*** | 52.67 | 25.61*** | 78.67 | 130.31*** | 50.60 | 18.14*** | 46.45 | 61.59*** | 33.08 | 360.31*** | 10.45 | 124.78*** | 2.93 | 245.11*** | 20.28 | 24.79*** | 29.60 | 312.69*** | 18.50 | 11.14*** |
| Yes | 12.85 | | 57.98 | | 54.59 | | 99.02 | | 67.23 | | 93.90 | | 57.84 | | 64.45 | | 52.11 | | 36.71 | | 11.39 | | 35.82 | | 64.92 | | 25.96 | |

***Note:*** *NA- data not available or cases not available; *** p<0.001, ** p<0.01, * p<0.05*

For peer review only

**Table 3:** Country specific bi-variate estimation on current use of e-cigarettes, GATS, 2011-2017

| | African Region | | | | Eastern Mediterranean | | European Region | | | |
|---|---|---|---|---|---|---|---|---|---|---|
| **Predictors** | **Ethiopia** | | **Senegal** | | **Qatar** | | **Greece** | | **Kazakhstan** | |
| | % | $\chi^2$ | % | $\chi^2$ | % | $\chi^2$ | % | $\chi^2$ | % | $\chi^2$ |
| **Gender** | | | | | | | | | | |
| Male | 1.79 | 1.10 | 0.89 | 0.04 | 2.51 | 12.51*** | 1.81 | 0.39 | 3.90 | 8.05** |
| Female | 1.34 | | 0.46 | | 0.48 | | 2.54 | | 2.09 | |
| **Age** | | | | | | | | | | |
| 15-24 | 2.25 | 0.86 | 1.89 | 3.29 | 0.93 | 4.07 | 0.00 | 12.39*** | 3.15 | 4.92 |
| 25-44 | 1.03 | | 0.34 | | 2.01 | | 2.99 | | 3.87 | |
| 45-64 | 3.03 | | 0.00 | | 2.55 | | 2.79 | | 1.99 | |
| above 64 | 0.00 | | 0.00 | | 0.00 | | 1.25 | | 0.27 | |
| **Residence** | | | | | | | | | | |
| Urban | 2.37 | 0.01 | 0.77 | 0.99 | NA | NA | 2.14 | 0.06 | 3.65 | 4.58** |
| Rural | 0.89 | | 0.52 | | NA | | 2.22 | | 2.02 | |
| **Education** | | | | | | | | | | |
| No formal education | 2.05 | 0.19 | 0.20 | 0.22 | 5.10 | 0.72 | 2.43 | 21.95*** | 2.13 | 2.85 |
| Completed primary | 1.69 | | 1.12 | | 1.50 | | 0.53 | | 2.32 | |
| Completed secondary | 1.55 | | 0.00 | | 1.64 | | 1.56 | | 2.81 | |
| Completed college/university | 1.24 | | 0.72 | | 1.93 | | 4.47 | | 3.81 | |
| **Occupation** | | | | | | | | | | |
| Non employed | 0.71 | 1.89 | 0.37 | 0.04 | 0.79 | 7.59*** | 1.23 | 14.58*** | 2.16 | 3.87** |
| Employed | 2.02 | | 1.02 | | 2.34 | | 3.29 | | 3.66 | |
| **Wealth Index** | | | | | | | | | | |
| Rich | 1.59 | 0.36 | 0.92 | 0.91 | NA | NA | 2.40 | 5.14** | 3.63 | 3.94 |
| Middle | 0.03 | | 0.00 | | NA | | NA | | 1.81 | |
| Poor | 2.03 | | 0.00 | | NA | | 1.36 | | 2.66 | |
| **Currently smoke tobacco** | | | | | | | | | | |
| No | 1.48 | 11.28*** | 0.72 | 2.30 | 0.37 | 173.69*** | 1.31 | 11.05*** | 0.83 | 87.43*** |
| Yes | 3.11 | | 0.90 | | 7.62 | | 3.36 | | 8.41 | |
| **Advertisements in stores** | | | | | | | | | | |
| No | 1.66 | 0.16 | 0.20 | 5.55** | 1.71 | 1.33 | 1.68 | 12.64*** | 3.02 | 1.03 |
| Yes | 0.00 | | 2.96 | | 2.92 | | 3.23 | | 3.68 | |
| **Advertisements on internet** | | | | | | | | | | |
| No | 1.65 | 0.13 | 0.77 | 0.15 | 1.83 | 0.03 | 2.11 | 2.33 | 3.17 | 0.15 |
| Yes | 0.00 | | 0.00 | | 1.90 | | 3.65 | | 2.55 | |

| | European Region (cont.) | | | | Regions of the Americas | | | |
|---|---|---|---|---|---|---|---|---|
| **Predictors** | **Russian Federation** | | **Ukraine** | | **Costa Rica** | | **Mexico** | |
| | % | $\chi^2$ | % | $\chi^2$ | % | $\chi^2$ | % | $\chi^2$ |
| **Gender** | | | | | | | | |
| Male | 6.29 | 80.19*** | 4.31 | 7.26*** | 3.10 | 4.68** | 2.62 | 9.49*** |
| Female | 2.55 | | 2.18 | | 2.18 | | 0.80 | |
| **Age** | | | | | | | | |
| 15-24 | 10.51 | 180.44*** | 7.60 | 63.71*** | 4.87 | 16.51*** | 3.57 | 24.53*** |
| 25-44 | 5.20 | | 3.86 | | 2.16 | | 1.28 | |
| 45-64 | 1.91 | | 1.44 | | 1.89 | | 0.66 | |
| above 64 | 0.54 | | 1.04 | | 0.45 | | 0.25 | |
| **Residence** | | | | | | | | |
| Urban | 4.86 | 38.16*** | 3.36 | 3.41 | 2.95 | 6.89*** | 1.85 | 1.76 |
| Rural | 2.8 | | 3.03 | | 1.58 | | 1.35 | |
| **Education** | | | | | | | | |
| No formal education | 0.00 | 4.37*** | 0.00 | 1.38 | 1.41 | 1.80 | 2.04 | 6.26 |
| Completed primary | 3.96 | | 0.37 | | 2.07 | | 0.72 | |
| Completed secondary | 4.24 | | 3.07 | | 3.75 | | 2.09 | |
| Completed college/university | 4.69 | | 3.88 | | 1.32 | | 1.59 | |
| **Occupation** | | | | | | | | |
| Non employed | 3.55 | 17.07*** | 2.09 | 9.40*** | 2.99 | 1.85 | 2.14 | 0.43 |
| Employed | 4.82 | | 4.18 | | 2.44 | | 1.57 | |
| **Wealth Index** | | | | | | | | |
| Rich | 4.82 | 16.79*** | 3.60 | 15.14 | 2.91 | 0.33 | 1.98 | 3.63 |
| Middle | 2.49 | | 3.96 | | 2.68 | | 1.98 | |
| Poor | 4.18 | | 1.28 | | 2.15 | | 0.49 | |
| **Currently smoke tobacco** | | | | | | | | |
| No | 1.77 | 219.38*** | 1.91 | 68.28*** | 1.69 | 117.02*** | 0.93 | 45.92*** |
| Yes | 9.33 | | 6.61 | | 8.97 | | 4.38 | |
| **Advertisements in stores** | | | | | | | | |
| No | 4.21 | 9.37*** | 2.91 | 14.41*** | 2.73 | 0.77 | 1.64 | 1.27 |
| Yes | 7.17 | | 5.35 | | 2.41 | | 2.12 | |
| **Advertisements on internet** | | | | | | | | |
| No | 3.99 | 24.26*** | 3.27 | 4.53** | 2.67 | 0.00 | 1.68 | 4.99** |
| Yes | 8.31 | | 3.52 | | 2.89 | | 2.43 | |

| | South-East Asia Region | | | | Western Pacific Region | | | | | |
|---|---|---|---|---|---|---|---|---|---|---|
| **Predictors** | **Indonesia** | | **India** | | **Malaysia** | | **Philippines** | | **Vietnam** | |
| | % | $\chi^2$ | % | $\chi^2$ | % | $\chi^2$ | % | $\chi^2$ | % | $\chi^2$ |
| **Gender** | | | | | | | | | | |
| Male | 3.24 | 6.94*** | 0.81 | 5.93** | 5.53 | 8.20*** | 3.70 | 25.79*** | 1.38 | 1.49 |
| Female | 0.00 | | 0.31 | | 0.00 | | 0.64 | | 0.77 | |
| **Age** | | | | | | | | | | |
| 15-24 | 1.31 | 1.54 | 0.51 | 6.59* | 3.94 | 6.80 | 1.84 | 1.53 | 0.66 | 2.20 |
| 25-44 | 2.47 | | 0.73 | | 2.27 | | 2.80 | | 1.26 | |
| 45-64 | 4.56 | | 0.61 | | 0.00 | | 2.24 | | 1.75 | |
| above 64 | 0.00 | | 1.51 | | 0.00 | | 2.42 | | 0.00 | |
| **Residence** | | | | | | | | | | |
| Urban | 2.75 | 0.35 | 0.93 | 0.73 | 4.60 | 4.24** | 3.07 | 6.99*** | 0.57 | 4.04** |
| Rural | 1.87 | | 0.28 | | 1.40 | | 1.15 | | 1.79 | |
| **Education** | | | | | | | | | | |
| No formal education | 0.00 | 1.88 | 1.09 | 1.43 | 6.44 | 3.76 | 0.00 | 1.29 | 0.00 | 3.04 |
| Completed primary | 4.16 | | 0.19 | | 4.36 | | 0.00 | | 1.63 | |
| Completed secondary | 2.12 | | 0.82 | | 4.81 | | 2.30 | | 1.03 | |
| Completed college/university | 2.85 | | 0.66 | | 1.59 | | 2.15 | | 1.42 | |
| **Occupation** | | | | | | | | | | |
| Non employed | 1.60 | 0.90 | 0.21 | 7.09*** | 0.49 | 3.42 | 1.63 | 5.48** | 0.00 | 4.78** |
| Employed | 2.87 | | 0.99 | | 5.62 | | 2.79 | | 1.55 | |
| **Wealth Index** | | | | | | | | | | |
| Rich | 2.27 | 1.05 | 0.60 | 2.52 | 3.30 | 0.25 | 2.91 | 4.81 | 1.02 | 0.09 |
| Middle | 4.21 | | 0.42 | | 6.57 | | 1.55 | | 1.66 | |
| Poor | 1.05 | | 1.06 | | 3.07 | | 1.09 | | 1.26 | |
| **Currently smoke tobacco** | | | | | | | | | | |
| No | 0.43 | 12.08*** | 0.36 | 59.09*** | 0.38 | 30.27*** | 0.59 | 80.01*** | 0.14 | 23.75*** |
| Yes | 4.25 | | 2.37 | | 10.42 | | 6.97 | | 3.67 | |
| **Advertisements in stores** | | | | | | | | | | |
| No | 2.32 | 1.05 | 0.6 | 0.6 | 3.71 | 0.07 | 2.29 | 0.87 | 1.01 | 0.48 |
| Yes | 2.59 | | 0.87 | | 4.64 | | 2.51 | | 2.09 | |
| **Advertisements on internet** | | | | | | | | | | |
| No | 2.65 | 1.49 | 0.66 | 0.04 | 3.85 | 0.75 | 2.05 | 11.94*** | 1.17 | 3.26** |
| Yes | 0.00 | | 0.75 | | 5.11 | | 4.83 | | 1.57 | |

***Note:*** *NA- data not available or cases not available; *** *** p<0.001, ** p<0.01, * p<0.05*

For peer review only

***Country specific multivariate logistic estimation***

Table 4 shows the country-specific multivariate logistic estimation of current use of e-cigarettes. In the European region, Greece, where awareness of e-cigarettes is the highest, females are 2.27 times more likely to use e-cigarettes than males. Females in Qatar (Eastern Mediterranean region), Russia (European region), Mexico (American region) and Philippines (Western Pacific region), are 0.42, 0.63, 0.37 and 0.44 times less likely respectively to use e-cigarettes than males. The odds of e-cigarette use show a gradual decrease with increasing age in the countries like Russia, Costa Rica, Mexico and Ukraine. It is also observed that there is a significant effect of place of residence on use of e-cigarettes in few of the countries, such as Ethiopia (AOR: 0.01, CI: 0.00, 0.15), Russia (AOR: 0.59, CI: 0.44, 0.80), Malaysia (AOR: 0.25, CI: 0.06, 0.95), Philippines (AOR: 0.40, CI: 0.21, 0.78) where people in rural areas are less likely to use e-cigarettes than those residing in the urban areas. Whereas in Vietnam, people from rural areas are 4.36 times more likely to smoke e-cigarettes. In terms of occupation, the employed people of Ukraine are 1.64 times more likely to use e-cigarettes than the unemployed people. Countries like Russia and Greece, which are in the European region, have shown significant variations in the use of e-cigarettes by level of education. Household's economic wellbeing (wealth index) have not shown any significant effect on e-cigarette usage for majority of the study countries. In contrast, poor people of Mexico (AOR: 0.17, CI: 0.07, 0.54) are less likely to use e-cigarettes than the rich. It is also found that the current tobacco smokers are more likely to use e-cigarettes than non-smokers across all the countries, except for Indonesia (South East Asia region), Senegal and Ethiopia (African region). Philippines (AOR: 2.96, CI: 1.41, 6.21) demonstrates a significant association between noticing advertisement on internet and usage of e-cigarettes at 1% level of significance.



For peer review only - http://bmjopen.bmj.com/site/about/guidelines.xhtml

**Table 4:** Country specific multivariable logistic regression for current use of e-cigarette, GATS, 2011-2017

| Predictors | African Region | | Eastern Mediterranean | European Region | | | | Regions of the Americas | | South-East Asia Region | | Western Pacific Region | | |
|---|---|---|---|---|---|---|---|---|---|---|---|---|---|---|
| | Ethiopia | Senegal | Qatar | Greece | Kazakhstan | Russian Federation | Ukraine | Costa Rica | Mexico | Indonesia | India | Malaysia | Philippines | Vietnam |
| | AOR (95% CI) | AOR (95% CI ) | AOR (95% CI ) | AOR (95% CI ) | AOR (95% CI ) | AOR (95% CI ) | AOR (95% CI ) | AOR (95% CI ) | AOR (95% CI ) | AOR (95% CI ) | AOR (95% CI ) | AOR (95% CI ) | AOR (95% CI ) | AOR (95% CI ) |
| **Gender** | | | | | | | | | | | | | | |
| Male® | 1 | 1 | 1 | 1 | 1 | 1 | 1 | 1 | 1 | 1 | 1 | 1 | 1 | 1 |
| Female | 0.38 (0.07,1.99) | 0.53 (0.04,6.33) | 0.42**(0.18,0.98) | 2.27*(1.12,4.6) | 1.7 (0.9,3.22) | 0.62**(0.46,0.85) | 0.83 (0.47,1.46) | 0.91 (0.48,1.72) | 0.37*(0.17,0.81) | NA | 0.84 (0.14,5.09) | NA | 0.43 (0.16,1.13) | [46.48]***(5.77,374.2) |
| **Age** | | | | | | | | | | | | | | |
| 15-24® | 1 | 1 | 1 | 1 | 1 | 1 | 1 | 1 | 1 | 1 | 1 | 1 | 1 | 1 |
| 25-44 | 0.16 (0.02,1.12) | 0.03 (0,1.84) | 1.18 (0.46,3.05) | 0.83 (0.21,3.25) | 0.53 (0.24,1.17) | 0.31***(0.22,0.44) | 0.31***(0.17,0.57) | 0.42**(0.23,0.75) | 0.35**(0.17,0.72) | 2.23 (0.46,10.69) | 0.47 (0.07,3.18) | 0.51 (0.08,3.23) | 1.04 (0.5,2.14) | 1.28 (0.33,4.99) |
| 45-64 | 0.50 (0,78.42) | NA | 1.47 (0.51,4.24) | 1.16 (0.28,4.88) | 0.29*(0.11,0.74) | 0.12***(0.08,0.19) | 0.14***(0.06,0.3) | 0.34**(0.17,0.7) | 0.17*(0.04,0.67) | 4.54 (1,20.61) | 0.35 (0.04,2.87) | NA | 0.87 (0.34,2.23) | 2.27 (0.52,9.94) |
| above 64 | NA | NA | NA | NA | 0.05**(0.01,0.42) | 0.05***(0.02,0.13) | 0.17**(0.05,0.58) | 0.10**(0.02,0.5) | 0.04*(0,0.51) | NA | 2.36 (0.32,17.4) | NA | 1.4 (0.23,8.47) | NA |
| **Residence** | | | | | | | | | | | | | | |
| Urban® | 1 | 1 | 1 | 1 | 1 | 1 | 1 | 1 | 1 | 1 | 1 | 1 | 1 | 1 |
| Rural | 0.01**(0.00,0.15) | 4.66 (0.34,64.51) | NA | 1.35 (0.63,2.91) | 0.82 (0.44,1.55) | 0.59**(0.44,0.80) | 0.89 (0.53,1.51) | 0.55 (0.3,1) | 0.9 (0.36,2.23) | 0.55 (0.15,2.06) | 0.19 (0.03,1.21) | 0.25*(0.06,0.95) | 0.40**(0.21,0.78) | 4.37*(1.08,17.73) |
| **Education** | | | | | | | | | | | | | | |
| No formal education® | 1 | 1 | 1 | 1 | 1 | 1 | 1 | 1 | 1 | 1 | 1 | 1 | 1 | 1 |
| Completed primary | 0.16 (0.00,9.09) | [38.65](0.13,11919.09) | 0.21 (0.03,1.38) | 0.17 (0.02,1.65) | 0.76 (0.17,3.48) | 0.52*(0.28,0.98) | 0.1*(0.01,0.82) | 2.18 (0.29,16.55) | 0.13 (0.02,1.02) | 0.95 (0.23,3.92) | 0.21 (0.03,1.68) | 0.1*(0.01,0.89) | NA | 0.56 (0.08,4.04) |
| Completed secondary | 0.59 (0.00,113.42) | NA | 0.23 (0.04,1.29) | 0.44 (0.09,2.25) | 0.78 (0.2,3.08) | 0.68*(0.5,0.92) | 0.65 (0.36,1.15) | 2.94 (0.4,21.91) | 0.24 (0.04,1.38) | 0.58 (0.19,1.82) | 1.38 (0.12,15.99) | 0.06*(0,0.71) | 0.75 (0.41,1.38) | 0.33 (0.09,1.19) |
| Completed college/university | 0.41 (0.01,17.31) | [67.97](0.65,7054.79) | 0.24 (0.05,1.2) | 1.08 (0.21,5.52) | 1.28 (0.31,5.29) | NA | NA | 1.31 (0.16,10.51) | 0.39 (0.06,2.32) | NA | 1.21 (0.12,12.55) | 0.03*(0,0.73) | NA | NA |
| **Occupation** | | | | | | | | | | | | | | |
| Non employed® | 1 | 1 | 1 | 1 | 1 | 1 | 1 | 1 | 1 | 1 | 1 | 1 | 1 | 1 |
| Employed | 9.16 (0.29,284.89) | 16.73 (0.28,999.11) | 1.01 (0.49,2.09) | 1.8 (0.84,3.84) | 0.96 (0.41,2.26) | 1 (0.71,1.41) | 1.64 (0.94,2.88) | 0.69 (0.4,1.19) | 0.58 (0.28,1.2) | 0.41 (0.11,1.49) | 3.89 (0.44,34.75) | 6.65 (0.49,89.96) | 0.95 (0.43,2.08) | NA |
| **Wealth Index** | | | | | | | | | | | | | | |
| Rich® | 1 | 1 | 1 | 1 | 1 | 1 | 1 | 1 | 1 | 1 | 1 | 1 | 1 | 1 |
| Middle | 0.08(0,2.42) | NA | NA | NA | 0.68 (0.26,1.8) | 0.57*(0.35,0.94) | 1.46 (0.77,2.75) | 0.85 (0.37,1.94) | 1.02 (0.48,2.19) | 1.88 (0.47,7.57) | 1.29 (0.15,11.28) | 0.92 (0.27,3.17) | 0.52 (0.23,1.17) | 0.71 (0.17,3.02) |
| Poor | [58.64]***(6.99,491.99) | NA | NA | 0.73 (0.26,2.06) | 0.86 (0.41,1.81) | 0.95 (0.69,1.31) | 0.54 (0.24,1.21) | 0.62 (0.32,1.2) | 0.17**(0.05,0.54) | 0.47 (0.05,4.19) | 4.66 (0.28,76.22) | 0.29 (0.01,6.79) | 0.42 (0.16,1.08) | 0.76 (0.08,7.49) |
| **Currently smoke tobacco** | | | | | | | | | | | | | | |
| No® | 1 | 1 | 1 | 1 | 1 | 1 | 1 | 1 | 1 | 1 | 1 | 1 | 1 | 1 |
| Yes | 2.38 (0.08,68.27) | 2.93 (0.22,38.99) | [17.68]***(6.56,47.67) | 2.26*(1.01,5.04) | [16.97]***(7.46,38.59) | 5.60***(4.15,7.54) | 3.62***(2.04,6.41) | 6.66***(3.83,11.59) | 4.8***(2.36,9.76) | 5.33 (0.99,28.55) | 4.86*(1.12,21.14) | 13.42*(1.31,137.7) | 12.19***(5.27,28.24) | [458.5]***(27.08,7763.37) |
| **Advertisements in stores** | | | | | | | | | | | | | | |
| No® | 1 | 1 | 1 | 1 | 1 | 1 | 1 | 1 | 1 | 1 | 1 | 1 | 1 | 1 |
| Yes | NA | [17.29]*(1.5,198.96) | 1.45 (0.63,3.36) | 1.82 (0.92,3.61) | 0.99 (0.45,2.2) | 1.32 (0.83,2.09) | 1.71 (0.93,3.16) | 0.63 (0.23,1.67) | 1.18 (0.55,2.54) | 1.3 (0.55,3.07) | 0.97 (0.22,4.25) | 0.78 (0.17,3.65) | 0.86 (0.48,1.54) | 1.76 (0.4,7.74) |
| **Advertisements on internet** | | | | | | | | | | | | | | |
| No® | 1 | 1 | 1 | 1 | 1 | 1 | 1 | 1 | 1 | 1 | 1 | 1 | 1 | 1 |
| Yes | NA | NA | 1.07 (0.36,3.17) | 1.63 (0.44,6.13) | 0.63 (0.22,1.74) | 1.41 (0.96,2.07) | 0.61 (0.23,1.63) | 1.14 (0.31,4.28) | 1.18 (0.51,2.74) | NA | 0.72 (0.05,10.21) | 0.46 (0.05,3.88) | 2.96**(1.41,6.21) | 2.05 (0.1,41.01) |

***Note**: NA- data not available or cases not available; ® Reference Category; *** p<0.001, ** p<0.01, * p<0.05; [ ] AOR is typically high as the odds of e-cigarette use in the reference population is too low compared the predictor population and thus not interpretable in general.*



For peer review only - http://bmjopen.bmj.com/site/about/guidelines.xhtml

***Predicted average prevalence of e-cigarettes use in the countries***

From the country-specific logistic regression (Table 5), we predicted the average national prevalence for the respective countries. When adjusted for the socio-economic and demographic factors we found that Malaysia carried the highest burden of e-cigarette use (39/1000 population) compared to Indonesia (29/1000 population) during 2011. Country like the Russian Federation carried a prevalence of 37/1000 population during 2016 whereas in Ethiopia, the prevalence was 16/1000 population in the same year. Qatar from the Eastern Mediterranean region and Greece from the European region showed almost an equal prevalence of e-cigarette use during 2013. On the other hand, Kazakhstan during 2014 and Ukraine during 2017 carried a prevalence of 33 and 30 persons per thousand population of e-cigarette use (Table 5).

**Table 5.** Estimates of country level average prevalence of e-cigarette use and the total number of users, GATS, 2011-2017

| WHO Region | Country | Survey Year | Total Population* | Average prevalence (predicted) |
|---|---|---|---|---|
| African Region | Ethiopia | 2016 | 103,603,501 | 0.016 |
| | Senegal | 2017 | 15,419,381 | 0.007 |
| Eastern Mediterranean | Qatar | 2013 | 2,336,574 | 0.018 |
| European Region | Greece | 2013 | 10,965,211 | 0.019 |
| | Kazakhstan | 2014 | 17,288,285 | 0.033 |
| | Russian Federation | 2016 | 144,342,396 | 0.037 |
| | Ukraine | 2017 | 44,831,135 | 0.030 |
| Region of the Americas | Costa Rica | 2015 | 4,847,804 | 0.024 |
| | Mexico | 2015 | 124,777,324 | 0.017 |
| South-east Asia Region | Indonesia | 2011 | 245,116,206 | 0.029 |
| | India® | 2017 | 1,338,658,835 | 0.012 |
| Western Pacific Region | Malaysia | 2011 | 28,650,955 | 0.039 |
| | Philippines | 2015 | 102,113,212 | 0.022 |
| | Vietnam | 2015 | 92,677,076 | 0.012 |

***Note:*** *®: Reference Country;*
* https://data.worldbank.org/indicator/SP.POP.TOTL?most_recent_year_desc=false;

**Discussion**

This study has examined the awareness of e-cigarette use, prevalence, and socio-demographic determinants utilizing the most recent round of the GATS surveys covering 14 selected countries. The developed nations like USA, UK, New Zealand, France, and Germany have their own individual surveys to provide general/population-specific estimates of e-cigarette use and awareness. It was estimated that the prevalence of e-cigarette use among USA middle and high school students increased from 3% in 2011 to 7% in 2012 [41]. According to the Local Tobacco Control Profiles for England annual report, there had been a significant increase in e-cigarette users (population aged ≥16) from 15% in 2014 to 19% in 2017 in UK. Additionally, it had also been found that use of e-cigarette was much more prevalent among the younger population (aged 16-24 years) than the older


For peer review only - http://bmjopen.bmj.com/site/about/guidelines.xhtml

population (aged ≥ 60 years) [42]. Quatremere et al., 2019 estimated that 4% of the population in Mainland France were e-cigarette user and 3% of them used it on a daily basis. Of these total users, 60% were men, and 58% had a bachelor's degree [43]. Eichler et al., 2016 using a population-based cross-sectional survey, conducted during 2016, found that 49% of males and 51% of females used e-cigarettes in Germany [44]. In New Zealand, 7% of the general population used e-cigarettes, with young persons (18-24 years) were more likely than those over 45 years [45]. Furthermore, independent of educational qualification, persons with higher income were more likely to use e-cigarettes than those with a moderate or lower income in New Zealand [45].

This study suggests that countries in the European and eastern Mediterranean regions have a higher awareness of e-cigarette than the countries of other regions. The awareness and current use of e-cigarettes are higher among urban population in almost all the countries studied. On the contrary, Greece and Vietnam show higher prevalence of awareness in the rural areas. It is possible that the exposure to advertisement, accessibility and availability of e-cigarettes may have led to higher e-cigarettes use in urban areas [46].

The countries of European region demonstrate higher prevalence of e-cigarette use with higher education level in the population. In addition, the wealthy population are more likely to use e-cigarettes. The prevalence of current e-cigarette use may be higher in Russian Federation (4.39%) because e-cigarettes are not covered under the tobacco control policy of Federal Law No. 15-FZ [47]. Therefore, the restriction on marketing and advertisement finds a policy gap [33]. Though e-cigarette regulation policies in Malaysia (3.94%) and in Ukraine (3.28%), puts a strict restrictions on distribution, importation, minimum age and in sales; yet the prevalence of e-cigarette use is moderately high in these two countries [48]. On the contrary, the least prevalence of e-cigarettes use in the countries like India (0.66%) and Vietnam (1.88%) is may be due to the ban on manufacturing, imports, sales, advertisement, and in distribution [48–50].

The statistical evidence from this study suggest that the urban population, males, young people (15-24), those with higher wealth scores and the higher educated individuals keep better knowledge of e-cigarettes across all the selected countries. Males have higher prevalence of e-cigarette use than the females, except in Greece. A study by Tzortzi et al., 2020 reported that both tobacco smoking and e-cigarette use among females are higher than males in Greece, which is attributable to better economic condition [51]. A study based on an online survey in USA, had found that male older adults use e-cigarettes as a medium to quit smoking while the initiation of e-cigarette use among females is influenced by their family and friends [52]. The present study has shown that e-cigarette use among the younger adults is high compared to the older adults across all the selected countries; which emphasizes the need of implementing new population specific policies to curb the use of nicotine at younger ages. Notably, the cross-country analysis does not show consistent association between use of e-cigarette and educational qualification across selected GATS countries. While, the wealthy population are more likely to use e-cigarettes.

The multivariate adjusted model also reveals the association of e-cigarettes with gender, age, resident type, and employment. Results also indicate that males have higher use of e-cigarette which could be due to quitting



For peer review only - http://bmjopen.bmj.com/site/about/guidelines.xhtml

tobacco smoking. In the counterpart, women's initiation of e-cigarette use is often restricted by social stigma and health concerns [53]. On the other hand, males purchase e-cigarettes independently while females rely on their peers, limiting their use [54,55]. In Costa Rica and Mexico, young adults use e-cigarettes, suggesting a relatively higher availability, affordability and moderate restrictive policies on nicotine vaping products [56]. Previous studies on tobacco smoking suggest that urban youths are more likely to use tobacco than their counterparts which may be due to urban accessibility, urban space, and advertisements [34,57]. Literatures also suggests that early employment is associated with daily use among youths [58]. Adolescents/youths who are either employed or receive more pocket money and supposedly enjoy economic independence are more likely to smoke and use other addictive substances [58,59]. The present study shows that the unemployed population has a lower prevalence of e-cigarette use, possibly due to a lack of discretionary income [60,61]. The findings are also in line with other studies explaining the association of the advertisement/promotions through various media that trigger the use of e-cigarette among young adults [54,62,63]. The use of the internet and social media for advertising, marketing, and ENDS promotion has expanded rapidly and concerns have been raised regarding deceptive health statements, claims on withdrawal effectiveness, and hence targeting the youths [64]. Although the ill-health effects of e-cigarette use are debated, it is still established that the aerosols of the majority of ENDS contain toxic chemicals that are hazardous to lung health and elevate the risk of cardiovascular diseases [65,66]. The promotion and marketing challenges of e-cigarettes revolve around the country's public experiences and tobacco control measures. In many countries, such as Australia, Brazil, Denmark, and others, e-cigarettes advertising and marketing are illegal [48]. On the other hand, sales in other countries are legal and authorized [67]. Interestingly, in United Kingdom (UK), despite a strong tobacco policy, e-cigarettes use is booming [14]. At the same time, countries like Costa Rica, Ecuador, Honduras, the Republic of Korea, Togo and Vietnam do not have regulations on sales of e-cigarettes [48,67]. Initially, the e-cigarettes manufacturers were independent and then transgressional tobacco companies came to venture into the market by creating a new form of nicotine consumers (young and non-smokers) pretending to curtail the smoking epidemic as a reputational tactic [68]. Although the present study is cross-sectional, it fails to capture the association of purchasing behavior and employment. Therefore, it generalizes the likelihood of economic stability and e-cigarette use across selected GATS countries.

An important strength of this study is that it provides important baseline e-cigarette prevalence data for multiple countries. As GATS data collections continue, the prevalence of e-cigarette use in each country can continue to be monitored and can be used to evaluate policy changes that occur. However, this study has some limitations. Firstly, we have considered only 14 countries in the temporal setting of 2011-2017. Thereafter, we could not incorporate the drastic changes in e-cigarette devices, brands, marketing between 2011-2017, which might have impacted the prevalence of e-cigarette use. Lastly, the outcome variables in this particular study are self-reported in nature, thus the study estimates are limited to self-reporting bias and system bias. The self-reporting bias among the individuals largely depends on their socio-economic and demographic characteristics, which has been controlled in the study. To address the system bias in the estimations, we utilized the country-specific survey weights to derive the estimates. Notably, the GATS surveys are popularly referred and used across countries to



For peer review only - http://bmjopen.bmj.com/site/about/guidelines.xhtml

provide the prevalence of tobacco and e-cigarette use in the surveyed population utilizing the self-reported information only. At the same time, according to the WHO-GATS survey protocol, the survey instruments were developed to collect the necessary information on e-cigarette awareness and its use and thus we are restricted to utilize these self-reported information in this study in the absence of any other relevant data for these countries. Nevertheless, despite a few lacunae, there are gaps in the knowledge of cross-country e-cigarette use and this study provides useful clues to policymakers for establishing effective measures for public health planning.

## Conclusion

The findings from this study show that e-cigarette use is widespread across these selected GATS countries, with a higher prevalence among young adults. At the same time, while exploring the country-specific patterns in the determinants of e-cigarette use; economic wellbeing and higher educational attainment have emerged as consistent predictors of e-cigarette use. With a cross-country analysis of GATS datasets, this study presents the most recent socio-economic and demographic patterns of e-cigarettes use and its determinants. In addition, this study identifies the sub-populations at higher risk of e-cigarette use so that policymakers and health professionals can be more assertive in their interventions.

## Declarations

### Ethics approval and consent to participate

*The data source is publicly available secondary data, and ethically approved by the survey authorities. Hence, no separate ethical approval required.*

### Consent for publication

*Not applicable.*

### Availability of data and materials

*The datasets used and/or analysed during the current study are available from the corresponding author on reasonable request.*

### Competing interests

*The authors declare that they have no competing interests.*

### Funding

*This research received no specific grant from any funding agency in the public, commercial, or not-for-profit sectors.*

### Author contributions

SK, SS, SD, and JK: Conceptualization, methodology, validation, formal analysis, investigation, writing



For peer review only - http://bmjopen.bmj.com/site/about/guidelines.xhtml

(original draft preparation). AC: Review and editing. EAB: Conceptualization, methodology, validation, formal analysis and writing (review and editing). BP: Review. All authors have read and approved the final manuscript.

**Acknowledgements**

*Not applicable*

For peer review only



For peer review only - http://bmjopen.bmj.com/site/about/guidelines.xhtml

## References


1. Caraballo RS, Jamal A, Nguyen KH, Kuiper NM, Arrazola RA. Electronic Nicotine Delivery System Use Among U.S. Adults, 2014. Am J Prev Med [Internet]. 2016 Feb [cited 2020 Dec 12];50(2):226–9. Available from: https://linkinghub.elsevier.com/retrieve/pii/S0749379715005528

2. Singh T, Arrazola RA, Corey CG, Husten CG, Neff LJ, Homa DM, et al. Tobacco Use Among Middle and High School Students — United States, 2011–2015. 2016;65(14):8.

3. Morean M, Butler E, Bold K, Kong G, Camenga D, Cavallo D. Preferring more e-cigarette flavors is associated with e-cigarette use frequency among adolescents but not adults. 2018;10.

4. Hartwell G, Thomas S, Egan M, Gilmore A, Petticrew M. E-cigarettes and equity: a systematic review of differences in awareness and use between sociodemographic groups. Tob Control [Internet]. 2017 Dec [cited 2020 Dec 12];26(e2):e85–91. Available from: https://tobaccocontrol.bmj.com/lookup/doi/10.1136/tobaccocontrol-2016-053222

5. U.S. Department of Health and Human Services. E-Cigarette Use Among Youth and Young Adults. A Report of the Surgeon General [Internet]. Centers for Disease Control and Prevention. 2017 [cited 2021 May 26]. Available from: https://www.cdc.gov/tobacco/data_statistics/sgr/e-cigarettes/index.htm

6. Mirbolouk M, Charkhchi P, Kianoush S, Uddin SMI, Orimoloye OA, Jaber R, et al. Prevalence and Distribution of E-Cigarette Use Among U.S. Adults: Behavioral Risk Factor Surveillance System, 2016. Ann Intern Med [Internet]. 2018 Oct 2 [cited 2021 Aug 22];169(7):429–38. Available from: https://www.acpjournals.org/doi/abs/10.7326/M17-3440

7. McMillen RC, Gottlieb MA, Winickoff JP. e-Cigarettes—The Roles of Regulation and Clinicians. JAMA Intern Med [Internet]. 2015 Oct 1 [cited 2021 May 26];175(10):1603–4. Available from: https://doi.org/10.1001/jamainternmed.2015.4436

8. Liu X, Lugo A, Davoli E, Gorini G, Pacifici R, Fernández E, et al. Electronic cigarettes in Italy: a tool for harm reduction or a gateway to smoking tobacco? Tob Control. 2020 Mar;29(2):148–52.

9. Bhatnagar A, Payne TJ, Robertson RM. Is There A Role for Electronic Cigarettes in Tobacco Cessation? J Am Heart Assoc. 2019 Jun 18;8(12):e012742.

10. Camenga DR, Kong G, Cavallo DA, Krishnan-Sarin S. Current and Former Smokers' Use of Electronic Cigarettes for Quitting Smoking: An Exploratory Study of Adolescents and Young Adults. Nicotine Tob Res Off J Soc Res Nicotine Tob. 2017 Nov 7;19(12):1531–5.

11. Hartmann-Boyce J, Begh R, Aveyard P. Electronic cigarettes for smoking cessation. BMJ [Internet]. 2018 Jan 17 [cited 2021 May 26];360:j5543. Available from: https://www.bmj.com/content/360/bmj.j5543

12. Mayer M, Reyes-Guzman C, Grana R, Choi K, Freedman ND. Demographic Characteristics, Cigarette Smoking, and e-Cigarette Use Among US Adults. JAMA Netw Open [Internet]. 2020 Oct 13 [cited 2021 May 26];3(10):e2020694. Available from: https://jamanetwork.com/journals/jamanetworkopen/fullarticle/2771440

13. Etter J-F. Electronic cigarettes: a survey of users. BMC Public Health [Internet]. 2010 May 4 [cited 2021 May 26];10(1):231. Available from: https://doi.org/10.1186/1471-2458-10-231

14. WHO Framework Convention on Tobacco Control, Conference of the Parties to the WHO Framework Convention on Tobacco Control, sixth session. (2014). Electronic nicotine delivery systems: Report by WHO. 2014 [cited 2021 May 26]; Available from: https://apps.who.int/iris/handle/10665/147110




For peer review only - http://bmjopen.bmj.com/site/about/guidelines.xhtml

15. National Academies of Sciences, Engineering, and Medicine, Health and Medicine Division, Board on Population Health and Public Health Practice, Committee on the Review of the Health Effects of Electronic Nicotine Delivery Systems. Public Health Consequences of E-Cigarettes [Internet]. Eaton DL, Kwan LY, Stratton K, editors. Washington (DC): National Academies Press (US); 2018 [cited 2021 May 26]. Available from: http://www.ncbi.nlm.nih.gov/books/NBK507171/

16. Suter MA, Mastrobattista J, Sachs M, Aagaard K. Is There Evidence for Potential Harm of Electronic Cigarette Use in Pregnancy? Birt Defects Res A Clin Mol Teratol [Internet]. 2015 Mar [cited 2021 May 26];103(3):186–95. Available from: https://www.ncbi.nlm.nih.gov/pmc/articles/PMC4830434/

17. Pepper JK, Ribisl KM, Brewer NT. Adolescents' interest in trying flavoured e-cigarettes. Tob Control [Internet]. 2016 Nov 1 [cited 2021 May 26];25(Suppl 2):ii62–6. Available from: https://tobaccocontrol.bmj.com/content/25/Suppl_2/ii62

18. Jones K, Salzman GA. The Vaping Epidemic in Adolescents. Mo Med [Internet]. 2020;117(1):56–8. Available from: https://www.ncbi.nlm.nih.gov/pmc/articles/PMC7023954/

19. Berry KM, Fetterman JL, Benjamin EJ, Bhatnagar A, Barrington-Trimis JL, Leventhal AM, et al. Association of Electronic Cigarette Use With Subsequent Initiation of Tobacco Cigarettes in US Youths. JAMA Netw Open [Internet]. 2019 Feb 1 [cited 2021 May 26];2(2):e187794. Available from: http://jamanetworkopen.jamanetwork.com/article.aspx?doi=10.1001/jamanetworkopen.2018.7794

20. Gentzke AS. Vital Signs: Tobacco Product Use Among Middle and High School Students — United States, 2011–2018. MMWR Morb Mortal Wkly Rep [Internet]. 2019 [cited 2021 May 27];68. Available from: https://www.cdc.gov/mmwr/volumes/68/wr/mm6806e1.htm

21. Simon P, Camenga DR, Morean ME, Kong G, Bold KW, Cavallo DA, et al. Socioeconomic status and adolescent e-cigarette use: The mediating role of e-cigarette advertisement exposure. Prev Med [Internet]. 2018 Jul 1 [cited 2021 May 27];112:193–8. Available from: https://www.sciencedirect.com/science/article/pii/S0091743518301300

22. Kock L, Shahab L, West R, Brown J. E-cigarette use in England 2014–17 as a function of socio-economic profile. Addiction [Internet]. 2019 [cited 2021 May 27];114(2):294–303. Available from: https://onlinelibrary.wiley.com/doi/abs/10.1111/add.14446

23. Rogers EM, Singhal A, Quinlan MM. Diffusion of Innovations [Internet]. An Integrated Approach to Communication Theory and Research. Routledge; 2014 [cited 2021 May 27]. Available from: https://www.taylorfrancis.com/https://www.taylorfrancis.com/chapters/edit/10.4324/9780203887011-36/diffusion-innovations-everett-rogers-arvind-singhal-margaret-quinlan

24. Thun M, Peto R, Boreham J, Lopez AD. Stages of the cigarette epidemic on entering its second century. Tob Control [Internet]. 2012 Mar [cited 2020 Dec 12];21(2):96–101. Available from: https://tobaccocontrol.bmj.com/lookup/doi/10.1136/tobaccocontrol-2011-050294

25. Pepper JK, Emery SL, Ribisl KM, Southwell BG, Brewer NT. Effects of advertisements on smokers' interest in trying e-cigarettes: the roles of product comparison and visual cues. 2014;6.

26. Duke JC, Lee YO, Kim AE, Watson KA, Arnold KY, Nonnemaker JM, et al. Exposure to Electronic Cigarette Television Advertisements Among Youth and Young Adults. PEDIATRICS [Internet]. 2014 Jul 1 [cited 2020 Dec 12];134(1):e29–36. Available from: http://pediatrics.aappublications.org/cgi/doi/10.1542/peds.2014-0269

27. Farrelly MC, Duke JC, Crankshaw EC, Eggers ME, Lee YO, Nonnemaker JM, et al. A Randomized Trial of the Effect of E-cigarette TV Advertisements on Intentions to Use E-cigarettes. Am J Prev Med [Internet]. 2015 Nov [cited 2020 Dec 12];49(5):686–93. Available from:



For peer review only - http://bmjopen.bmj.com/site/about/guidelines.xhtml

https://linkinghub.elsevier.com/retrieve/pii/S0749379715002330

28. Robertson L, Cameron C, McGee R, Marsh L, Hoek J. Point-of-sale tobacco promotion and youth smoking: a meta-analysis. Tob Control [Internet]. 2016 Dec [cited 2020 Dec 12];25(e2):e83–9. Available from: https://tobaccocontrol.bmj.com/lookup/doi/10.1136/tobaccocontrol-2015-052586

29. WHO report on the global tobacco epidemic, 2015: Rising Taxes on Tobacco [Internet]. WHO. World Health Organization; 2015 [cited 2021 May 26]. Available from: http://www.who.int/tobacco/global_report/2015/en/

30. WHO report on the global tobacco epidemic, 2017: Monitoring tobacco use and prevention policies [Internet]. WHO. World Health Organization; 2017 [cited 2021 May 26]. Available from: http://www.who.int/tobacco/global_report/2017/en/

31. Report of the seventh session of the Conference of the Parties to the WHO Framework Convention on Tobacco Control Delhi, India, 7–12 November 2016 [Internet]. WHO. World Health Organization; 2016 [cited 2021 May 26]. Available from: http://www.who.int/fctc/cop/sessions/cop7/cop7-summary/en/

32. WHO report on the global tobacco epidemic, 2019: Offer help to quit tobacco use [Internet]. 2019 [cited 2021 May 26]. Available from: https://www.who.int/teams/health-product-and-policy-standards/access-to-assistive-technology-medical-devices/health-promotion

33. Lagasse LP, Grant AS, Minosa MKR, Kennedy RD, Cohen JE. Availability and advertising of electronic cigarettes in two Russian cities following implementation of comprehensive tobacco advertising restrictions. Tob Induc Dis [Internet]. 2020 [cited 2021 May 27];18(January):4. Available from: https://jhu.pure.elsevier.com/en/publications/availability-and-advertising-of-electronic-cigarettes-in-two-russ

34. Noland M, Rayens MK, Wiggins AT, Huntington-Moskos L, Rayens EA, Howard T, et al. Current Use of E-Cigarettes and Conventional Cigarettes Among US High School Students in Urban and Rural Locations: 2014 National Youth Tobacco Survey. Am J Health Promot [Internet]. 2018 Jun [cited 2020 Dec 12];32(5):1239–47. Available from: http://journals.sagepub.com/doi/10.1177/0890117117719621

35. Palipudi KM, Mbulo L, Morton J, Mbulo L, Bunnell R, Blutcher-Nelson G, et al. Awareness and Current Use of Electronic Cigarettes in Indonesia, Malaysia, Qatar, and Greece: Findings From 2011–2013 Global Adult Tobacco Surveys. Nicotine Tob Res [Internet]. 2016 Apr [cited 2020 Dec 12];18(4):501–7. Available from: https://academic.oup.com/ntr/article-lookup/doi/10.1093/ntr/ntv081

36. King BA, Mirza SA, Babb SD, for the GATS Collaborating Group. A cross-country comparison of secondhand smoke exposure among adults: findings from the Global Adult Tobacco Survey (GATS). Tob Control [Internet]. 2013 Jul [cited 2020 Dec 12];22(4):e5–e5. Available from: https://tobaccocontrol.bmj.com/lookup/doi/10.1136/tobaccocontrol-2012-050582

37. Kostova D, Chaloupka FJ, Yurekli A, Ross H, Cherukupalli R, Andes L, et al. A cross-country study of cigarette prices and affordability: evidence from the Global Adult Tobacco Survey. Tob Control [Internet]. 2014 Jan [cited 2020 Dec 12];23(1):e3–e3. Available from: https://tobaccocontrol.bmj.com/lookup/doi/10.1136/tobaccocontrol-2011-050413

38. Morton J, Song Y, Fouad H, Awa FE, Abou El Naga R, Zhao L, et al. Cross-country comparison of waterpipe use: nationally representative data from 13 low and middle-income countries from the Global Adult Tobacco Survey (GATS). Tob Control [Internet]. 2014 Sep [cited 2020 Dec 12];23(5):419–27. Available from: https://tobaccocontrol.bmj.com/lookup/doi/10.1136/tobaccocontrol-2012-050841

39. Rutstein SO, Johnson K. The DHS wealth index. 2004 Aug 1 [cited 2021 Sep 2]; Available from: https://dhsprogram.com/publications/publication-cr6-comparative-reports.cfm



For peer review only - http://bmjopen.bmj.com/site/about/guidelines.xhtml

40. Rutstein SO. Steps to constructing the new DHS Wealth Index [Internet]. 2015 [cited 2021 Sep 2]. Available from: https://dhsprogram.com/programming/wealth%20index/Steps_to_constructing_the_new_DHS_Wealth_Index.pdf

41. Corey C, Wang B, Johnson SE, Apelberg B, Husten C, King BA, et al. Notes from the Field: Electronic cigarette use among middle and high school students - United States, 2011-2012. Morb Mortal Wkly Rep [Internet]. 2013 [cited 2021 Sep 1];62(35):729–30. Available from: https://jhu.pure.elsevier.com/en/publications/notes-from-the-field-electronic-cigarette-use-among-middle-and-hi-5

42. Cornish D, Brookman A, Horton M, Scanlon S. Adult smoking habits in the UK: 2017. Office For National Statistics: Office For National Statistics. [Internet]. 2018 [cited 2021 Aug 31]. Available from: https://www.ons.gov.uk/peoplepopulationandcommunity/healthandsocialcare/drugusealcoholandsmoking/datasets/ecigaretteuseingreatbritain

43. Quatremère G, Pasquereau A, Guignard R, Andler R, Richard JB, Nguyen Thanh V. Use and perceptions of electronic cigarette by the adult population in France in 2017. Eur J Public Health [Internet]. 2019 Nov 1 [cited 2021 Sep 1];29(Supplement_4). Available from: https://doi.org/10.1093/eurpub/ckz186.626

44. Eichler M, Blettner M, Singer S. The Use of E-Cigarettes. Dtsch Ärztebl Int [Internet]. 2016 Dec [cited 2021 Sep 1];113(50):847–54. Available from: https://www.ncbi.nlm.nih.gov/pmc/articles/PMC5273587/

45. Li J, Bullen C, Newcombe R, Walker N, Walton D. The use and acceptability of electronic cigarettes among New Zealand smokers. N Z Med J. 2013 May 1;126(1375):48–57.

46. Collins L, Glasser AM, Abudayyeh H, Pearson JL, Villanti AC. E-Cigarette Marketing and Communication: How E-Cigarette Companies Market E-Cigarettes and the Public Engages with E-cigarette Information. Nicotine Tob Res [Internet]. 2019 Jan 1 [cited 2021 May 26];21(1):14–24. Available from: https://doi.org/10.1093/ntr/ntx284

47. Russian Federation - Federal Law No.15-FZ of 23 February 2013 on Health Protection from Exposure to Environmental Tobacco Smoke and the Consequences of Tobacco Consumption. [Internet]. 2013 [cited 2021 May 27]. Available from: http://www.ilo.org/dyn/natlex/natlex4.detail?p_lang=en&p_isn=96223&p_country=RUS&p_count=1

48. Kennedy RD, Awopegba A, León ED, Cohen JE. Global approaches to regulating electronic cigarettes. Tob Control [Internet]. 2017 Jul 1 [cited 2021 May 27];26(4):440–5. Available from: https://tobaccocontrol.bmj.com/content/26/4/440

49. Dyer O. India bans e-cigarettes by executive order. BMJ [Internet]. 2019 Sep 19 [cited 2021 May 27];366:l5649. Available from: https://www.bmj.com/content/366/bmj.l5649

50. Jha P. Smoking cessation and e-cigarettes in China and India. BMJ [Internet]. 2019 Oct 18 [cited 2021 May 27];367:l6016. Available from: https://www.bmj.com/content/367/bmj.l6016

51. Tzortzi A, Kapetanstratak M, Evangelopoulou V, Behrakis P. Smoking in Greece Where we stand in 2020. 2020;33(2):9. Available from: http://www.pneumon.org/arpil-june-2020-vol-33-issue-2/newsid789/790

52. Piñeiro B, Correa JB, Simmons VN, Harrell PT, Menzie NS, Unrod M, et al. Gender differences in use and expectancies of e-cigarettes: Online survey results. Addict Behav [Internet]. 2016 Jan [cited 2020 Dec 12];52:91–7. Available from: https://linkinghub.elsevier.com/retrieve/pii/S0306460315300174

53. Bauhoff S, Montero A, Scharf D. Perceptions of e-cigarettes: a comparison of adult smokers and non-



For peer review only - http://bmjopen.bmj.com/site/about/guidelines.xhtml

smokers in a Mechanical Turk sample. Am J Drug Alcohol Abuse [Internet]. 2017 May 4 [cited 2020 Dec 12];43(3):311–23. Available from: https://www.tandfonline.com/doi/full/10.1080/00952990.2016.1207654

54. Kong G, Morean ME, Cavallo DA, Camenga DR, Krishnan-Sarin S. Sources of Electronic Cigarette Acquisition among Adolescents in Connecticut. Tob Regul Sci. 2017 Jan 1;3(1):10–6.

55. Kong G, Kuguru KE, Krishnan-Sarin S. Gender Differences in U.S. Adolescent E-Cigarette Use. Curr Addict Rep [Internet]. 2017 Dec 1 [cited 2021 May 27];4(4):422–30. Available from: https://doi.org/10.1007/s40429-017-0176-5

56. Gravely S, Driezen P, Ouimet J, Quah ACK, Cummings KM, Thompson ME, et al. Prevalence of awareness, ever-use and current use of nicotine vaping products (NVPs) among adult current smokers and ex-smokers in 14 countries with differing regulations on sales and marketing of NVPs: cross-sectional findings from the ITC Project. Addiction [Internet]. 2019 [cited 2021 May 26];114(6):1060–73. Available from: https://onlinelibrary.wiley.com/doi/abs/10.1111/add.14558

57. Pesko MF, Robarts AMT. Adolescent Tobacco Use in Urban Versus Rural Areas of the United States: The Influence of Tobacco Control Policy Environments. J Adolesc Health [Internet]. 2017 Jul [cited 2020 Dec 12];61(1):70–6. Available from: https://linkinghub.elsevier.com/retrieve/pii/S1054139X17300629

58. Wu L, Schlenger W, Galvin D. The relationship between employment and substance use among students aged 12 to 17*1. J Adolesc Health [Internet]. 2003 Jan [cited 2020 Dec 12];32(1):5–15. Available from: https://linkinghub.elsevier.com/retrieve/pii/S1054139X02004470

59. Scragg R, Laugesen M, Robinson E. Cigarette smoking, pocket money and socioeconomic status: results from a national survey of 4th form students in 2000. 2002;115(1158):8.

60. Griesbach D, Amos A, Currie C. Adolescent smoking and family structure in Europe. Soc Sci Med [Internet]. 2003 Jan [cited 2020 Dec 12];56(1):41–52. Available from: https://linkinghub.elsevier.com/retrieve/pii/S027795360200014X

61. Darling H, Reeder AI, McGee R, Williams S. Brief report: Disposable income, and spending on fast food, alcohol, cigarettes, and gambling by New Zealand secondary school students. J Adolesc [Internet]. 2006 Oct [cited 2020 Dec 12];29(5):837–43. Available from: https://linkinghub.elsevier.com/retrieve/pii/S014019710600056X

62. Ashford K, Rayens E, Wiggins AT, Rayens MK, Fallin A, Sayre MM. Advertising exposure and use of e-cigarettes among female current and former tobacco users of childbearing age. Public Health Nurs [Internet]. 2017 [cited 2021 May 27];34(5):430–6. Available from: https://onlinelibrary.wiley.com/doi/abs/10.1111/phn.12334

63. Dai H, Hao J. Exposure to Advertisements and Susceptibility to Electronic Cigarette Use Among Youth. J Adolesc Health [Internet]. 2016 Dec [cited 2020 Dec 12];59(6):620–6. Available from: https://linkinghub.elsevier.com/retrieve/pii/S1054139X16301598

64. WHO. E-cigarettes are harmful to health [Internet]. 2020 [cited 2021 May 27]. Available from: https://www.who.int/news/item/05-02-2020-e-cigarettes-are-harmful-to-health

65. MacDonald A, Middlekauff HR. Electronic cigarettes and cardiovascular health: what do we know so far? Vasc Health Risk Manag [Internet]. 2019 Jun 21 [cited 2021 Sep 2];15:159–74. Available from: https://www.dovepress.com/electronic-cigarettes-and-cardiovascular-health-what-do-we-know-so-far-peer-reviewed-fulltext-article-VHRM

66. Buchanan ND, Grimmer JA, Tanwar V, Schwieterman N, Mohler PJ, Wold LE. Cardiovascular risk of



For peer review only - http://bmjopen.bmj.com/site/about/guidelines.xhtml

electronic cigarettes: a review of preclinical and clinical studies. Cardiovasc Res [Internet]. 2020 Jan 1 [cited 2021 Sep 2];116(1):40–50. Available from: https://doi.org/10.1093/cvr/cvz256

67. Institute for Global Tobacco Control. Country Laws Regulating E-cigarettes: A Policy Scan. Baltimore, MD: Johns Hopkins Bloomberg School of Public Health [Internet]. Global Tobacco Control - Learning from the Experts. 2015 [cited 2021 May 26]. Available from: https://www.globaltobaccocontrol.org/e-cigarette_policyscan

68. Peeters S, Gilmore AB. Understanding the emergence of the tobacco industry's use of the term tobacco harm reduction in order to inform public health policy. Tob Control [Internet]. 2015 Mar [cited 2020 Dec 12];24(2):182–9. Available from: https://tobaccocontrol.bmj.com/lookup/doi/10.1136/tobaccocontrol-2013-051502

For peer review only



For peer review only - http://bmjopen.bmj.com/site/about/guidelines.xhtml

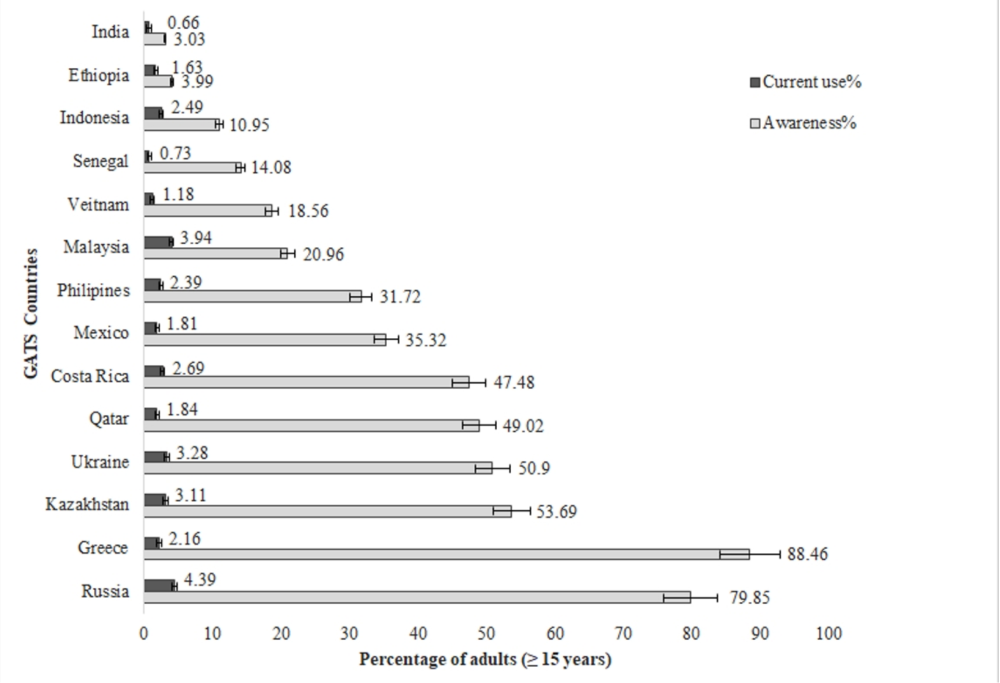


The prevalence of e-cigarettes usage and awareness across selected GATS countries

525x358mm (236 x 236 DPI)

BMJ Open

# Age, gender and socio-economic patterns of awareness and usage of e-cigarettes across selected WHO region countries: evidence from the Global Adult Tobacco Survey

| | |
|---|---|
| Journal: | *BMJ Open* |
| Manuscript ID | bmjopen-2022-070419.R1 |
| Article Type: | Original research |
| Date Submitted by the Author: | 13-Dec-2022 |
| Complete List of Authors: | Kundu, Sampurna; Jawaharlal Nehru University, Centre of Social Medicine and Community Health; International Institute for Population Sciences, Department of Survey Research & Data Analytics<br>Shaw, Subhojit; International Institute for Population Sciences, Department of Population and Development<br>Khan, Junaid; International Institute for Population Sciences, Department of Population and Development<br>Chattopadhyay, Aparajita; International Institute for Population Sciences, Department of Population and Development<br>Baptista, Emerson; El Colegio de Mexico AC<br>Paswan, Balram; International Institute for Population Sciences |
| <b>Primary Subject Heading</b>: | Smoking and tobacco |
| Secondary Subject Heading: | Public health |
| Keywords: | PUBLIC HEALTH, SOCIAL MEDICINE, Health policy < HEALTH SERVICES ADMINISTRATION & MANAGEMENT |
| | |

SCHOLARONE™ Manuscripts

*I, the Submitting Author has the right to grant and does grant on behalf of all authors of the Work (as defined in the below author licence), an exclusive licence and/or a non-exclusive licence for contributions from authors who are: i) UK Crown employees; ii) where BMJ has agreed a CC-BY licence shall apply, and/or iii) in accordance with the terms applicable for US Federal Government officers or employees acting as part of their official duties; on a worldwide, perpetual, irrevocable, royalty-free basis to BMJ Publishing Group Ltd ("BMJ") its licensees and where the relevant Journal is co-owned by BMJ to the co-owners of the Journal, to publish the Work in this journal and any other BMJ products and to exploit all rights, as set out in our licence.*

*The Submitting Author accepts and understands that any supply made under these terms is made by BMJ to the Submitting Author unless you are acting as an employee on behalf of your employer or a postgraduate student of an affiliated institution which is paying any applicable article publishing charge ("APC") for Open Access articles. Where the Submitting Author wishes to make the Work available on an Open Access basis (and intends to pay the relevant APC), the terms of reuse of such Open Access shall be governed by a Creative Commons licence – details of these licences and which Creative Commons licence will apply to this Work are set out in our licence referred to above.*

*Other than as permitted in any relevant BMJ Author's Self Archiving Policies, I confirm this Work has not been accepted for publication elsewhere, is not being considered for publication elsewhere and does not duplicate material already published. I confirm all authors consent to publication of this Work and authorise the granting of this licence.*

For peer review only

# Age, gender and socio-economic patterns of awareness and usage of e-cigarettes across selected WHO region countries: evidence from the Global Adult Tobacco Survey

Sampurna Kundu[1], Subhojit Shaw[2], Junaid Khan[2], Aparajita Chattopadhyay[2], Emerson Augusto Baptista[3,*], Balram Paswan[2]

[1] Jawaharlal Nehru University, New Delhi, Delhi 110067, India.

[2] International Institute for Population Sciences, Deonar, Mumbai 400088, India.

[3] El Colegio de México, CEDUA, Mexico City 14110, México.

* Correspondence to Prof. Aparajita Chattopadhyay, Email id: aparajita@iipsindia.ac.in

**Word count:** 4464

## Abstract

**Objectives** The purpose of this study is to describe awareness and prevalence of e-cigarette use by demographic and socio-economic characteristics of the study population (≥15 years) in selected 14 Global Adult Tobacco Survey (GATS) countries.

**Design** Cross-sectional.

**Setting** 14 countries.

**Participants** Country-specific surveyed population (≥15 years) constitute the study sample. Individuals were selected using multi-stage cluster sampling design.

Primary and secondary outcome measures We selected 14 countries from six different WHO regions where GATS was conducted in different years during 2011-2017. Using bivariate cross-tabulation, we estimated the prevalence of e-cigarette use by socio-economic and demographic characteristics. In addition, country specific multivariable logistic regression was estimated to examine the pattern and determinants of e-cigarette use in the respective countries.

**Results** Among the selected GATS countries, awareness of e-cigarette was highest in Greece (88%), whereas it was observed to be lowest in India (3%). TOn the other hand, the prevalence of e-cigarette use was highest in Russia (4%) and lowest in India (0.7%). Across all countries, the prevalence of e-cigarette use is was higher among males except Greece. A few European and American region countries have reported high use of e-cigarette among younger age group (15-24). In general, the logistic estimation showed that those who are of better socio-economic condition (rich, higher educated, resides in urban areas) are more likely to use e-cigarette than their counterparts. With the unconventional pattern, females in Greece were 2.3 times (AOR: 2.27; p<0.01) more likely to use e-cigarettes than males. When adjusted for all other factors, tThe age-associated estimates of adjusted odds ratio informed no consistent pattern of likelihood across the selected countries.

**Conclusions** This study shows that the urban population, males, young people aged 15-24, people with higher wealth index scores and the educated population have the awareness a better knowledge of e-cigarette across all the countries. As e-cigarette use is much more prevalent among young adults, a community level surveillance is needed to reduce the burden of e-cigarette use among males and other risky groups. As e-cigarette use is much more prevalent among young adults, their behaviors also need to be closely monitored at different levels.

**Keywords**: E-cigarette; GATS; Gender; Global Health; Public Policy; Tobacco Smokers.



For peer review only - http://bmjopen.bmj.com/site/about/guidelines.xhtml

**Strengths and limitations of this study**

- This study provides an important baseline e-cigarette prevalence data for multiple countries.
- This cross-country study explores the factors affecting adolescent and adult (≥ 15 years) behavior of e-cigarettes usage in 14 selected GATS countries.
- Among the current tobacco smokers, the likelihood of e-cigarette use is higher across all the study countries.
- A limitation to this study is that the estimates of e-cigarette awareness and its use are based on self-reported information.
- As GATS data collections continue, the prevalence of e-cigarette use in each country can continue to be monitored and thus can be used to evaluate the existing policies over time.

For peer review only

## Introduction

The use of e-cigarettes has become common among youths and there are concerns that widespread use may lead to future epidemic[1–3]. Subsequently, the popularity and use of e-cigarettes have increased over the years[4]. E-cigarettes and other battery-powered vaporizers were first launched in China, 2003 and later on entered the US market in 2007[5]. Surprisingly, during 2016, an estimated 54.6% of the e-cigarette users were also conventional cigarette smokers among US adults[6]. The use of e-cigarettes have evolved as a means of reducing the harmful effects of smoking while still delivering nicotine. E-cigarettes vaporize a liquid that contains nicotine for inhalation without the need of burning leaf materials. Despite of its widespread adoption and high public exposure, there remains a disparity in the diffusion of e-cigarette awareness among different socio-demographic groups[4].

There is no consensus in the previous literatures on the long-term effect or benefits of quitting smoking with e-cigarettes[7]. A few studies suggest that e-cigarettes can serve as a gateway for subsequent tobacco smoking[8–11]. According to a cross-sectional study in the USA, e-cigarette use is low among former smokers than the current adult smokers [12]. A study conducted by the United States Food and Drug Administration (FDA) revealed that e-cigarettes contain carcinogens, nitrosamines, diethylene glycol and other chemicals [13]. These chemicals have adverse effects on children, adolescents, and pregnant mothers, contributing to cardiovascular diseases [14–16]. E-cigarettes are also available in varied flavors, making them more attractive and appealing to the youths [17]. For older adult smokers, e-cigarettes possess a beneficial health transition; however, amateur young adults who use e-cigarettes have a potential health risk. While nicotine itself is not a carcinogen, malignant diseases and neurodegeneration are suspected to result from nicotine [14]. A legitimate concern among the children is that if children (non-smokers) develops a nicotine addiction, they may start smoking cigarettes [18]. A recent study has suggested that young adults who use e-cigarettes had higher odds of conventional smoking initiation[19]. Furthermore, a report published by the National Academies of Sciences, Engineering, and Medicine (2018) had found evidence of an increase in the risk of tobacco smoking due to e-cigarette use among young adults [15]. Hence, this poses a major public health challenge that requires strict regulation to access e-cigarettes.

A study conducted in the USA had shown that e-cigarette usage rates increased from 1.5% to 20.8% among youths during 2011-2018 [20]. The use of e-cigarettes among youth adolescents has raised concerns about a new generation's lifelong addiction. Some previous literatures showed an increase in the use of e-cigarettes among the youth, especially those in the higher economic sections [21,22]. The diffusion and innovation theory by Everett Rogers, proposed in 1962, suggested that innovations are first appreciated by the upper class, followed by others [23,24]. E-cigarette use mimics low nicotine, reduces tar exposure, and is more aesthetically appealing than other forms of smoking, making it more attractive alternative with a higher rate of adaptation [25]. Therefore, an understanding of e-cigarette usage and its proximate determinants factors across different nations is critical. A handful of studies found that the likelihood of e-cigarette use was higher in the event of exposure



For peer review only - http://bmjopen.bmj.com/site/about/guidelines.xhtml

to advertisements/promotions [26–28]. Since 2014, the Institute for Global Tobacco Control (IGTC), under Johns Hopkins Bloomberg School of Public Health, has been working globally across 120 nations to measure tobacco advertisement, promotion and evaluate the e-cigarette policy. In 2015, the WHO and World Bank Group called on WHO member countries to increase tobacco and related product taxes to prevent youths from initiating tobacco use [29]. In subsequent years, through numerous policies and programs, the WHO offered to help fight the tobacco epidemic [30]. In collaboration, WHO member countries have developed mechanisms for tobacco cessation support. At the same time, the amount and quality of scientific evidence have not been sufficient to determine whether e-cigarettes may help most smokers to quit or prevent tobacco smoking [31]. According to the WHO, member countries should regulate e-cigarettes to avoid promotion and intake by non-smokers, pregnant women, and youths [32].

A systematic review of literatures by Hartwell et al., 2017, has shown that the usage of e-cigarettes is higher among young adults, in higher socio-economic classes, and among individuals with a higher level of education [4]. The use of e-cigarettes is on the rise and a significant amount of research has been undertaken from a small selection of high-income countries [1,2,8,12,33,34]. In general, the use of e-cigarette is increasing among young adults as well as among the general adult population across many countries. GATS surveys in its member countries lately introduced a set of questions to collect the self-reported information on e-cigarette use and its awareness. Given the list of GATS survey countries, there exists no holistic research exploring the socio-economic and demographic determinants of e-cigarette awareness and its use. In this context, the present cross-country study explores the factors affecting adolescent and adult (population over 15 years) behavior of e-cigarettes usage, employing the most recent round of the Global Adult Tobacco Survey (GATS) datasets. From a socio-political perspective, this study is a timely contribution to identify the sub-population at higher risk of using e-cigarettes.

**Data**

Global Adult Tobacco Survey (GATS), an integral part of the Global Tobacco Surveillance System (GTSS), is a nationally representative household survey started in 2008. The survey collects specific information on tobacco use and tracks key tobacco control indicators among non-institutionalized adults 15 years of age or older in GATS countries. GATS uses country-specific stratified multi-stage cluster sampling designs in which probability proportional to size (PPS) random selection methods are used to successively choose clusters in one or more steps to secure ample coverage of the target population. The sample of households is chosen in two or more stages, with sampling units in the first or second stage being well-defined geopolitical areas within the country. These areas are then randomly selected from a complete list of enumeration areas having no more than 250 households. At the final stage, households were surveyed from the randomly selected areas. From the selected household, one individual aged 15 years or older was randomly chosen to participate in the survey. The collection of information was carried out using electronic handheld devices. The overall response rate ranged



For peer review only - http://bmjopen.bmj.com/site/about/guidelines.xhtml

from 64.4% to 98.5% across the selected counties in the study. For the first time since 2011, GATS introduced the questions on e-cigarette awareness and the current use of e-cigarettes [35]. Table 1 shows the GATS datasets being included in the study.

**Table 1**. Description of the GATS dataset included in the study

| WHO Regions | Country | Survey Year | Households Surveyed | Individuals Interviewed | Response Rate (%) |
|---|---|---|---|---|---|
| African Region | Ethiopia | 2016 | 10,875 | 10,150 | 93.4 |
| | Senegal | 2017 | 4,514 | 4,347 | 97.0 |
| Eastern Mediterranean | Qatar | 2013 | 8,571 | 8,398 | 98.5 |
| European Region | Greece | 2013 | 6,600 | 4,359 | 69.6 |
| | Kazakhstan | 2014 | 4,611 | 4,425 | 96.7 |
| | Russian Federation | 2016 | 11,764 | 11,458 | 98.2 |
| | Ukraine | 2017 | 14,800 | 8,298 | 64.4 |
| Region of the Americas | Costa Rica | 2015 | 9,680 | 8,607 | 89.2 |
| | Mexico | 2015 | 17,765 | 14,664 | 82.7 |
| South-east Asia Region | Indonesia | 2011 | 8,994 | 8,305 | 94.3 |
| | India | 2017 | 84,047 | 74,037 | 92.9 |
| Western pacific Region | Malaysia | 2011 | 5,112 | 4,250 | 85.3 |
| | Philippines | 2015 | 13,963 | 11,644 | 88.4 |
| | Vietnam | 2015 | 9,514 | 8,996 | 95.8 |

***Note:*** Compiled by authors

The surveys across the countries tracked down the awareness of e-cigarettes at different survey time points. In the survey questionnaire, e-cigarettes have been described as "Electronic cigarettes include any product that uses batteries or other methods to produce a vapor which contains nicotine. The description also includes various other names such as e-cigarette, vape-pen, e-shisha, e-pipes". The respondents were then asked "Have you ever heard of e-cigarettes?" Those who responded 'yes' were considered to be aware of e-cigarettes. Those individuals who were aware of e-cigarettes were further asked to respond if he/she had currently used e-cigarettes on a daily basis, less than daily, or not at all. Those who responded as 'daily' or 'less than daily' were considered current users of e-cigarettes. In this study, we selected those fourteen countries that collected the specific information on e-cigarette use and its awareness in their most recent round of the GATS surveys (Table 1) [36–38].

### *Outcome variable*

The key outcome variable of this study was the respondent's current e-cigarette use status and was defined as a dichotomous (yes/no) variable. The variable was defined as '1' if the respondent answered as currently using e-cigarettes on 'daily' or 'less than daily' basis and '0' otherwise.

### *Independent variables*

Among the independent variables, the study utilized various socio-economic and demographic characteristics of the study population such as gender, age, residence, wealth index, education and occupation. According to the sample distribution, age was recoded into four age-groups: 15-24, 25-44, 45-64, and above 64. Education



For peer review only - http://bmjopen.bmj.com/site/about/guidelines.xhtml

was categorized as: no formal education/less than primary, completed primary/less than secondary, completed secondary or completed high school, and completed college or university or above. The ‘occupation’ variable included two categories, ‘employed’ and ‘non-employed/unemployed’.

Wealth quintile variable has been computed in this study to measure the economic status of the respondents, which is commonly used in the cross sectional surveys [39]. Authors have followed the standard DHS framework to compute the wealth variable using principal component analysis (PCA) [40]. While this is a common computation method of creating wealth index variable where households are given scores based on the number and kinds of consumer goods it own, ranging from electricity connectivity, flash toilet, fixed telephone, cell telephone, television, radio, car, refrigerator, scooter or motor cycle, washing machine, computer or laptop, internet connection, air conditioner and electric fan. Then each individual from the same household are ranked by the scores and the distribution was equally divided into five quintile categories. of the bottom 20% of the population is identified as poorest, next 20% as poorer, and likewise the top 20% is identified as the richest. In the present study we have further recoded the wealth quintile variable into three categories, such as – poor, middle and rich.

The current tobacco use status was assessed by the question ‘Do you currently smoke tobacco on a daily basis, less than daily, or not at all?’. People who responded ‘daily’ or ‘less than daily’ were considered as current tobacco smokers (coded 1) and ‘not at all’ were considered non-smokers (coded 0). The tobacco products used included manufactured cigarettes, cigars, pipes, hand-rolled cigarettes, kreteks, and water pipes.

The study used two types of noticing to advertisements- in stores and on the internet. The store advertisement noticing was measured by the question ‘In the past 30 days, have you noticed any advertisements or signs promoting cigarettes in stores where cigarettes are sold?’, and, similarly, internet advertisement noticing was measured by the question ‘In the past 30 days, have you noticed any advertisements or signs promoting cigarettes on the internet?’ Respondents who said ‘Yes’ were the individuals to notice the advertisements (coded 1), and coded ‘No’ as ‘0’ otherwise.

**Statistical Analysis**

For each selected country, we calculated the prevalence of current use of e-cigarettes, as well as the percentage of awareness about e-cigarettes. Further, we used bivariate analysis in the form of chi-square test of independence and cross-tabulations as well. To examine the socio-economic and demographic determinants of e-cigarette use in the population of the respective countries, we employed country specific multivariable logistic regression. The general equation of the logistic equation is as follows-

$$logit\left(\frac{p_i}{1-p_i}\right) = \alpha + \beta_1(gender_i) + \beta_2(age_i) + \beta_3(residence_i) + \beta_4(education_i) + \beta_5(occupation_i) + \beta_6(wealth\ index_i) + \beta_7(CST_i) + \beta_8(Ad_{storesi}) + \beta_9(Ad_{interneti}) + \varepsilon_i$$



For peer review only - http://bmjopen.bmj.com/site/about/guidelines.xhtml

Here $\alpha$ is the intercept, $\beta_j s$ are the coefficients and $\varepsilon_i$ is the error component. CST refers to current smoking of tobacco, 'Ad_stores' refers to the variable of noticing advertisements in stores and similarly 'Ad_internet' refers to noticing advertisements on internet.

Once the logistic model was fitted for a particular country, we estimated the predicted prevalence from the model fit which gave the average prevalence of e-cigarette use after adjusting for all other socio-economic and demographic factors for that country. The 'logit' package in STATA provides different features for post-estimation. There are different post-estimation commands available from the same package and we used the 'predict' command which by default estimates the probability of the positive outcome (Y=1). This 'predict' command generates a new variable to store the estimated probability for each of the study individual/entity. Thus, for each of the country specific fitted logistic model, we estimated the average probability of the positive outcome, which is actually the average probability of using e-cigarette in the study population and hence the average predicted prevalence of e-cigarette use in a particular country. Sample weights ('gatsweight' in the survey dataset) have been used throughout the analysis. STATA version 14.1 was used to analyse the datasets.

**Patient and Public Involvement**

No patient involved

**Results**

***Awareness and current usage of e-cigarettes***

Figure 1 shows awareness and current usage of e-cigarettes across selected countries. The percentage of people with awareness of e-cigarettes is highest in Greece (88.46%), followed by Russia (79.84%) and Kazakhstan (53.69%), that is, countries located in the European region. At the other extreme, India is the country with lowest percentage of people with awareness of e-cigarettes (3.03%), followed by Ethiopia (3.99%), and Indonesia (10.95%). Hence, overall, the countries belonging to the European region shows a maximum level of awareness in comparison to countries of other WHO regions.

**Figure 1**. The prevalence of e-cigarettes usage and awareness across selected GATS countries

**[Fig 1 about here]**

*Compiled by authors, Source: https://nccd.cdc.gov/GTSSDataSurveyResources/Ancillary/DataReports.aspx?CAID=2*

Table 2 also shows that awareness of e-cigarettes is higher among males and younger people. In addition, e-cigarette awareness is higher among those with more education (completed high school and higher), higher wealth index scores, noticing advertisement, living in urban areas, employed, and those who are currently smoking tobacco. In summary, the chi-square test (Table 2) shows that there is a significant association between awareness of e-cigarettes and gender, age, place of residence, educational level, occupation, wealth quintile,



For peer review only - http://bmjopen.bmj.com/site/about/guidelines.xhtml

current use of tobacco smoking and noticing of advertisement for almost all GATS countries studied. Exceptions are observed for Qatar, in Eastern Mediterranean, where there is no significant association of awareness with noticing advertising in stores.

Regarding the prevalence of current use of e-cigarettes, it is found to be generally low in majority of the selected GATS countries. The highest percentage of current usage of e-cigarettes is observed in Russia (4.39%), followed by Costa Rica (2.69%). On the other hand, countries like India (0.66%) and Senegal (0.73%) in the South-East Asia and African regions respectively shows lowest current use(Table 3). For Senegal (African region) and Vietnam (West Pacific region), although the prevalence is higher among men, there is no significant association observed between gender and current use of e-cigarettes . Few countries in European and American regions reflect high usage among the younger age groups (15-24 age): Russia (10.5%), Ukraine (7.6%), and Costa Rica (4.87%). In the American region, Costa Rica has the higher usage overall, but it is significantly associated with gender, age, residence and current tobacco smoking only. In addition, few countries show high usage among older age groups (64 age and above): Philippines (2.42%), India (1.51%), and Greece (1.25%), which are in the Western Pacific, South-east Asia and European region, respectively (Table 3). In terms of the age groups, there are significant differences observed in current e-cigarette use across the countries of Costa Rica, Greece, Mexico, and Ukraine. At the same time, except Greece and Russia (European region), none of the countries had any significant association of current use of e-cigarettes with educational level, although people with higher levels of education make more use of e-cigarettes (completed college or university, Greece: 4.5% and Russia: 4.7%). In all countries, the prevalence of current e-cigarette use is observed to be higher among those who smoke tobacco than the non-smokers. It is also found that current usage of smoking tobacco has a significant association with current use of e-cigarettes in the population across all the countries, except for Senegal (users: 0.9%, non-users: 0.72%) (Table 3).

For peer review only

**Table 2:** Country specific bi-variate estimation on awareness of e-cigarette use, GATS, 2011-2017

| | African Region | | | | Eastern Mediterranean | | European Region | | | | | | | | Regions of the Americas | | | | South-East Asia Region | | | | Western Pacific Region | | | | | |
|---|---|---|---|---|---|---|---|---|---|---|---|---|---|---|---|---|---|---|---|---|---|---|---|---|---|---|---|---|
| **Predictors** | **Ethiopia** | | **Senegal** | | **Qatar** | | **Greece** | | **Kazakhstan** | | **Russian Federation** | | **Ukraine** | | **Costa Rica** | | **Mexico** | | **Indonesia** | | **India** | | **Malaysia** | | **Philippines** | | **Vietnam** | |
| | % | $\chi^2$ | % | $\chi^2$ | % | $\chi^2$ | % | $\chi^2$ | % | $\chi^2$ | % | $\chi^2$ | % | $\chi^2$ | % | $\chi^2$ | % | $\chi^2$ | % | $\chi^2$ | % | $\chi^2$ | % | $\chi^2$ | % | $\chi^2$ | % | $\chi^2$ |
| **Gender** | | | | | | | | | | | | | | | | | | | | | | | | | | | | |
| Male | 5.23 | 58.90*** | 18.70 | 74.21*** | 62.80 | 835.79*** | 93.43 | 129.39*** | 64.06 | 174.80*** | 86.28 | 315.84*** | 58.04 | 216.63*** | 51.98 | 58.53*** | 40.93 | 193.02*** | 16.83 | 307.30*** | 4.14 | 313.67*** | 29.00 | 181.44*** | 36.36 | 122.65*** | 25.53 | 352.65*** |
| Female | 2.74 | | 9.73 | | 33.81 | | 83.67 | | 44.36 | | 74.52 | | 44.96 | | 42.94 | | 30.15 | | 5.09 | | 1.87 | | 12.43 | | 27.10 | | 12.01 | |
| **Age** | | | | | | | | | | | | | | | | | | | | | | | | | | | | |
| 15-24 | 2.96 | 19.69*** | 12.56 | 33.56*** | 48.78 | 78.31*** | 93.73 | 803.79*** | 59.56 | 350.55*** | 91.45 | 2400.00*** | 63.09 | 921.62*** | 51.93 | 207.56*** | 43.91 | 379.98*** | 14.24 | 141.08*** | 3.75 | 165.95*** | 24.11 | 139.43*** | 35.75 | 250.85*** | 20.97 | 143.86*** |
| 25-44 | 5.24 | | 16.30 | | 50.17 | | 95.22 | | 62.24 | | 92.26 | | 61.72 | | 51.72 | | 37.74 | | 12.53 | | 3.54 | | 26.17 | | 35.30 | | 21.64 | |
| 45-64 | 3.64 | | 14.81 | | 49.52 | | 35.21 | | 47.18 | | 80.17 | | 51.20 | | 43.56 | | 30.51 | | 7.89 | | 2.12 | | 13.06 | | 26.61 | | 15.64 | |
| above 64 | 3.96 | | 5.09 | | 25.83 | | 62.17 | | 19.54 | | 44.11 | | 24.30 | | 28.91 | | 15.21 | | 1.72 | | 0.91 | | 7.57 | | 12.54 | | 7.90 | |
| **Residence** | | | | | | | | | | | | | | | | | | | | | | | | | | | | |
| Urban | 8.27 | 176.61*** | 24.22 | 362.19*** | NA | NA | 90.43 | 73.74*** | 63.42 | 225.18*** | 81.98 | 211.83*** | 55.89 | 247.33*** | 51.80 | 248.05*** | 41.17 | 1100.00*** | 15.34 | 181.09*** | 5.12 | 683.54*** | 23.05 | 38.55*** | 43.83 | 576.93*** | 27.51 | 195.41*** |
| Rural | 2.62 | | 3.97 | | NA | | 83.25 | | 40.97 | | 73.46 | | 39.58 | | 35.21 | | 13.71 | | 6.53 | | 1.93 | | 15.55 | | 21.11 | | 14.00 | |
| **Education** | | | | | | | | | | | | | | | | | | | | | | | | | | | | |
| No formal education | 2.42 | 348.46*** | 2.97 | 782.24*** | 15.95 | 328.34*** | 49.85 | 809.60*** | 36.84 | 221.97*** | 33.44 | 1100.00*** | 49.25 | 503.06*** | 28.48 | 462.22*** | 9.40 | 1500.00*** | 1.40 | 575.35*** | 0.70 | 2300.00*** | 6.56 | 292.71*** | 13.16 | 840.25*** | 4.11 | 725.71*** |
| Completed primary | 3.26 | | 26.34 | | 39.68 | | 74.55 | | 38.70 | | 56.29 | | 15.07 | | 37.84 | | 21.22 | | 5.74 | | 2.13 | | 13.21 | | 21.25 | | 9.57 | |
| Completed secondary | 5.07 | | 35.98 | | 48.53 | | 94.43 | | 56.43 | | 79.08 | | 49.57 | | 50.04 | | 42.39 | | 16.08 | | 4.27 | | 23.27 | | 23.10 | | 20.76 | |
| Completed college/university | 13.99 | | 59.68 | | 56.52 | | 96.50 | | 64.02 | | 89.33 | | 61.28 | | 68.05 | | 59.20 | | 29.41 | | 10.07 | | 43.28 | | 50.30 | | 39.90 | |
| **Occupation** | | | | | | | | | | | | | | | | | | | | | | | | | | | | |
| Non employed | 2.35 | 25.56*** | 12.07 | 25.57*** | 36.00 | 604.03*** | 82.72 | 291.99*** | 41.10 | 309.02*** | 65.19 | 1400.00*** | 41.29 | 538.77*** | 40.94 | 184.19*** | 30.38 | 247.09*** | 8.89 | 28.85*** | 2.66 | 47.21*** | 15.58 | 105.77*** | 28.08 | 38.85*** | 16.93 | 16.32*** |
| Employed | 5.59 | | 16.23 | | 59.14 | | 96.58 | | 65.11 | | 90.47 | | 61.69 | | 54.73 | | 40.02 | | 12.16 | | 3.37 | | 25.19 | | 34.06 | | 19.15 | |
| **Wealth Index** | | | | | | | | | | | | | | | | | | | | | | | | | | | | |
| Rich | 7.76 | 269.44*** | 25.74 | 248.38*** | NA | NA | 93.57 | 398.44*** | 66.06 | 305.87*** | 86.09 | 614.97*** | 61.24 | 731.61*** | 54.79 | 271.09*** | 48.67 | 1500.00*** | 18.49 | 432.58*** | 5.86 | 1400.00*** | 27.46 | 185.19*** | 44.73 | 847.92*** | 31.08 | 615.73*** |
| Middle | 2.66 | | 9.47 | | NA | | NA | | 50.71 | | 75.80 | | 46.04 | | 42.99 | | 25.96 | | 8.07 | | 1.79 | | 19.35 | | 25.94 | | 16.54 | |
| Poor | 2.85 | | 2.60 | | NA | | 74.90 | | 37.72 | | 69.26 | | 32.09 | | 37.77 | | 14.80 | | 3.15 | | 1.28 | | 9.02 | | 15.97 | | 8.11 | |
| **Currently smoke tobacco** | | | | | | | | | | | | | | | | | | | | | | | | | | | | |
| No | 3.75 | 70.39*** | 13.66 | 7.17*** | 44.41 | 529.69*** | 83.93 | 219.58*** | 48.35 | 184.62*** | 74.97 | 493.53*** | 46.72 | 263.43*** | 45.00 | 194.59*** | 31.46 | 592.80*** | 7.75 | 163.63*** | 2.88 | 33.89*** | 17.60 | 101.71*** | 29.43 | 63.30*** | 16.91 | 67.99*** |
| Yes | 10.10 | | 21.49 | | 82.68 | | 95.79 | | 72.16 | | 91.07 | | 65.02 | | 72.79 | | 55.03 | | 16.94 | | 4.24 | | 32.11 | | 39.52 | | 24.25 | |
| **Advertisements in stores** | | | | | | | | | | | | | | | | | | | | | | | | | | | | |
| No | 3.95 | 19.15*** | 12.44 | 64.13*** | 48.91 | 0.59 | 85.38 | 120.96*** | 53.75 | 0.01*** | 79.33 | 37.97*** | 50.01 | 39.73*** | 46.47 | 23.91*** | 33.08 | 109.49*** | 7.93 | 124.73*** | 2.60 | 327.46*** | 19.55 | 22.53*** | 28.85 | 66.40*** | 17.15 | 99.34*** |
| Yes | 15.38 | | 31.35 | | 49.93 | | 96.26 | | 53.27 | | 89.22 | | 56.49 | | 57.00 | | 39.22 | | 14.54 | | 7.17 | | 27.00 | | 35.94 | | 33.49 | |
| **Advertisements on internet** | | | | | | | | | | | | | | | | | | | | | | | | | | | | |
| No | 3.96 | 11.31*** | 13.62 | 86.62*** | 48.20 | 6.90*** | 88.13 | 16.81*** | 52.67 | 25.61*** | 78.67 | 130.31*** | 50.60 | 18.14*** | 46.45 | 61.59*** | 33.08 | 360.31*** | 10.45 | 124.78*** | 2.93 | 245.11*** | 20.28 | 24.79*** | 29.60 | 312.69*** | 18.50 | 11.14*** |
| Yes | 12.85 | | 57.98 | | 54.59 | | 99.02 | | 67.23 | | 93.90 | | 57.84 | | 64.45 | | 52.11 | | 36.71 | | 11.39 | | 35.82 | | 64.92 | | 25.96 | |

***Note:*** *NA- data not available or cases not available; *** p<0.001, ** p<0.01, * p<0.05*



For peer review only

**Table 3:** Country specific bi-variate estimation on current use of e-cigarettes, GATS, 2011-2017

| | African Region | | | | Eastern Mediterranean | | European Region | | | | | | | | Regions of the Americas | | | | South-East Asia Region | | | | Western Pacific Region | | | | | |
|---|---|---|---|---|---|---|---|---|---|---|---|---|---|---|---|---|---|---|---|---|---|---|---|---|---|---|---|---|
| **Predictors** | **Ethiopia** | | **Senegal** | | **Qatar** | | **Greece** | | **Kazakhstan** | | **Russian Federation** | | **Ukraine** | | **Costa Rica** | | **Mexico** | | **Indonesia** | | **India** | | **Malaysia** | | **Philippines** | | **Vietnam** | |
| | % | $\chi^2$ | % | $\chi^2$ | % | $\chi^2$ | % | $\chi^2$ | % | $\chi^2$ | % | $\chi^2$ | % | $\chi^2$ | % | $\chi^2$ | % | $\chi^2$ | % | $\chi^2$ | % | $\chi^2$ | % | $\chi^2$ | % | $\chi^2$ | % | $\chi^2$ |
| **Gender** | | | | | | | | | | | | | | | | | | | | | | | | | | | | |
| Male | 1.79 | 1.10 | 0.89 | 0.04 | 2.51 | 12.51*** | 1.81 | 0.39 | 3.90 | 8.05** | 6.29 | 80.19*** | 4.31 | 7.26*** | 3.10 | 4.68** | 2.62 | 9.49*** | 3.24 | 6.94*** | 0.81 | 5.93** | 5.53 | 8.20*** | 3.70 | 25.79*** | 1.38 | 1.49 |
| Female | 1.34 | | 0.46 | | 0.48 | | 2.54 | | 2.09 | | 2.55 | | 2.18 | | 2.18 | | 0.80 | | 0.00 | | 0.31 | | 0.00 | | 0.64 | | 0.77 | |
| **Age** | | | | | | | | | | | | | | | | | | | | | | | | | | | | |
| 15-24 | 2.25 | 0.86 | 1.89 | 3.29 | 0.93 | 4.07 | 0.00 | 12.39*** | 3.15 | 4.92 | 10.51 | 180.44*** | 7.60 | 63.71*** | 4.87 | 16.51*** | 3.57 | 24.53*** | 1.31 | 1.54 | 0.51 | 6.59* | 3.94 | 6.80 | 1.84 | 1.53 | 0.66 | 2.20 |
| 25-44 | 1.03 | | 0.34 | | 2.01 | | 2.99 | | 3.87 | | 5.20 | | 3.86 | | 2.16 | | 1.28 | | 2.47 | | 0.73 | | 2.27 | | 2.80 | | 1.26 | |
| 45-64 | 3.03 | | 0.00 | | 2.55 | | 2.79 | | 1.99 | | 1.91 | | 1.44 | | 1.89 | | 0.66 | | 4.56 | | 0.61 | | 0.00 | | 2.24 | | 1.75 | |
| above 64 | 0.00 | | 0.00 | | 0.00 | | 1.25 | | 0.27 | | 0.54 | | 1.04 | | 0.45 | | 0.25 | | 0.00 | | 1.51 | | 0.00 | | 2.42 | | 0.00 | |
| **Residence** | | | | | | | | | | | | | | | | | | | | | | | | | | | | |
| Urban | 2.37 | 0.01 | 0.77 | 0.99 | NA | NA | 2.14 | 0.06 | 3.65 | 4.58** | 4.86 | 38.16*** | 3.36 | 3.41 | 2.95 | 6.89*** | 1.85 | 1.76 | 2.75 | 0.35 | 0.93 | 0.73 | 4.60 | 4.24** | 3.07 | 6.99*** | 0.57 | 4.04** |
| Rural | 0.89 | | 0.52 | | NA | | 2.22 | | 2.02 | | 2.8 | | 3.03 | | 1.58 | | 1.35 | | 1.87 | | 0.28 | | 1.40 | | 1.15 | | 1.79 | |
| **Education** | | | | | | | | | | | | | | | | | | | | | | | | | | | | |
| No formal education | 2.05 | 0.19 | 0.20 | 0.22 | 5.10 | 0.72 | 2.43 | 21.95*** | 2.13 | 2.85 | 0.00 | 4.37*** | 0.00 | 1.38 | 1.41 | 1.80 | 2.04 | 6.26 | 0.00 | 1.88 | 1.09 | 1.43 | 6.44 | 3.76 | 0.00 | 1.29 | 0.00 | 3.04 |
| Completed primary | 1.69 | | 1.12 | | 1.50 | | 0.53 | | 2.32 | | 3.96 | | 0.37 | | 2.07 | | 0.72 | | 4.16 | | 0.19 | | 4.36 | | 0.00 | | 1.63 | |
| Completed secondary | 1.55 | | 0.00 | | 1.64 | | 1.56 | | 2.81 | | 4.24 | | 3.07 | | 3.75 | | 2.09 | | 2.12 | | 0.82 | | 4.81 | | 2.30 | | 1.03 | |
| Completed college/university | 1.24 | | 0.72 | | 1.93 | | 4.47 | | 3.81 | | 4.69 | | 3.88 | | 1.32 | | 1.59 | | 2.85 | | 0.66 | | 1.59 | | 2.15 | | 1.42 | |
| **Occupation** | | | | | | | | | | | | | | | | | | | | | | | | | | | | |
| Non employed | 0.71 | 1.89 | 0.37 | 0.04 | 0.79 | 7.59*** | 1.23 | 14.58*** | 2.16 | 3.87** | 3.55 | 17.07*** | 2.09 | 9.40*** | 2.99 | 1.85 | 2.14 | 0.43 | 1.60 | 0.90 | 0.21 | 7.09*** | 0.49 | 3.42 | 1.63 | 5.48** | 0.00 | 4.78** |
| Employed | 2.02 | | 1.02 | | 2.34 | | 3.29 | | 3.66 | | 4.82 | | 4.18 | | 2.44 | | 1.57 | | 2.87 | | 0.99 | | 5.62 | | 2.79 | | 1.55 | |
| **Wealth Index** | | | | | | | | | | | | | | | | | | | | | | | | | | | | |
| Rich | 1.59 | 0.36 | 0.92 | 0.91 | NA | NA | 2.40 | 5.14** | 3.63 | 3.94 | 4.82 | 16.79*** | 3.60 | 15.14 | 2.91 | 0.33 | 1.98 | 3.63 | 2.27 | 1.05 | 0.60 | 2.52 | 3.30 | 0.25 | 2.91 | 4.81 | 1.02 | 0.09 |
| Middle | 0.03 | | 0.00 | | NA | | NA | | 1.81 | | 2.49 | | 3.96 | | 2.68 | | 1.98 | | 4.21 | | 0.42 | | 6.57 | | 1.55 | | 1.66 | |
| Poor | 2.03 | | 0.00 | | NA | | 1.36 | | 2.66 | | 4.18 | | 1.28 | | 2.15 | | 0.49 | | 1.05 | | 1.06 | | 3.07 | | 1.09 | | 1.26 | |
| **Currently smoke tobacco** | | | | | | | | | | | | | | | | | | | | | | | | | | | | |
| No | 1.48 | 11.28*** | 0.72 | 2.30 | 0.37 | 173.69*** | 1.31 | 11.05*** | 0.83 | 87.43*** | 1.77 | 219.38*** | 1.91 | 68.28*** | 1.69 | 117.02*** | 0.93 | 45.92*** | 0.43 | 12.08*** | 0.36 | 59.09*** | 0.38 | 30.27*** | 0.59 | 80.01*** | 0.14 | 23.75*** |
| Yes | 3.11 | | 0.90 | | 7.62 | | 3.36 | | 8.41 | | 9.33 | | 6.61 | | 8.97 | | 4.38 | | 4.25 | | 2.37 | | 10.42 | | 6.97 | | 3.67 | |
| **Advertisements in stores** | | | | | | | | | | | | | | | | | | | | | | | | | | | | |
| No | 1.66 | 0.16 | 0.20 | 5.55** | 1.71 | 1.33 | 1.68 | 12.64*** | 3.02 | 1.03 | 4.21 | 9.37*** | 2.91 | 14.41*** | 2.73 | 0.77 | 1.64 | 1.27 | 2.32 | 1.05 | 0.6 | 0.6 | 3.71 | 0.07 | 2.29 | 0.87 | 1.01 | 0.48 |
| Yes | 0.00 | | 2.96 | | 2.92 | | 3.23 | | 3.68 | | 7.17 | | 5.35 | | 2.41 | | 2.12 | | 2.59 | | 0.87 | | 4.64 | | 2.51 | | 2.09 | |
| **Advertisements on internet** | | | | | | | | | | | | | | | | | | | | | | | | | | | | |
| No | 1.65 | 0.13 | 0.77 | 0.15 | 1.83 | 0.03 | 2.11 | 2.33 | 3.17 | 0.15 | 3.99 | 24.26*** | 3.27 | 4.53** | 2.67 | 0.00 | 1.68 | 4.99** | 2.65 | 1.49 | 0.66 | 0.04 | 3.85 | 0.75 | 2.05 | 11.94*** | 1.17 | 3.26** |
| Yes | 0.00 | | 0.00 | | 1.90 | | 3.65 | | 2.55 | | 8.31 | | 3.52 | | 2.89 | | 2.43 | | 0.00 | | 0.75 | | 5.11 | | 4.83 | | 1.57 | |

***Note:*** *NA- data not available or cases not available; *** *** p<0.001, ** p<0.01, * p<0.05*

***Country specific multivariate logistic estimation***

Table 4 shows the country-specific multivariate logistic estimation of current use of e-cigarettes. In the European region, Greece, where awareness of e-cigarettes is the highest, females are 2.27 times more likely to use e-cigarettes than males. Females in Qatar (Eastern Mediterranean region), Russia (European region), Mexico (American region) and Philippines (Western Pacific region), are 0.42, 0.63, 0.37 and 0.44 times less likely respectively to use e-cigarettes than males. The odds of e-cigarette use show a gradual decrease with increasing age in the countries like Russia, Costa Rica, Mexico and Ukraine. It is also observed that there is a significant effect of place of residence on use of e-cigarettes in few of the countries, such as Ethiopia (AOR: 0.01, CI: 0.00, 0.15), Russia (AOR: 0.59, CI: 0.44, 0.80), Malaysia (AOR: 0.25, CI: 0.06, 0.95), Philippines (AOR: 0.40, CI: 0.21, 0.78) where people in rural areas are less likely to use e-cigarettes than those residing in the urban areas. Whereas in Vietnam, people from rural areas are 4.36 times more likely to smoke e-cigarettes. In terms of occupation, the employed people of Ukraine are 1.64 times more likely to use e-cigarettes than the unemployed people. Countries like Russia and Greece, which are in the European region, have shown significant variations in the use of e-cigarettes by level of education. Household's economic wellbeing (wealth index) have not shown any significant effect on e-cigarette usage for majority of the study countries. In contrast, poor people of Mexico (AOR: 0.17, CI: 0.07, 0.54) are less likely to use e-cigarettes than the rich. It is also found that the current tobacco smokers are more likely to use e-cigarettes than non-smokers across all the countries, except for Indonesia (South East Asia region), Senegal and Ethiopia (African region). Philippines (AOR: 2.96, CI: 1.41, 6.21) demonstrates a significant association between noticing advertisement on internet and usage of e-cigarettes at 1% level of significance.



For peer review only - http://bmjopen.bmj.com/site/about/guidelines.xhtml

For peer review only

**Table 4:** Country specific multivariable logistic regression for current use of e-cigarette, GATS, 2011-2017

| | African Region | | Eastern Mediterranean | European Region | | | | Regions of the Americas | | South-East Asia Region | | Western Pacific Region | | |
|---|---|---|---|---|---|---|---|---|---|---|---|---|---|---|
| **Predictors** | **Ethiopia** | **Senegal** | **Qatar** | **Greece** | **Kazakhstan** | **Russian Federation** | **Ukraine** | **Costa Rica** | **Mexico** | **Indonesia** | **India** | **Malaysia** | **Philippines** | **Vietnam** |
| | AOR (95% CI) | AOR (95% CI ) | AOR (95% CI ) | AOR (95% CI ) | AOR (95% CI ) | AOR (95% CI ) | AOR (95% CI ) | AOR (95% CI ) | AOR (95% CI ) | AOR (95% CI ) | AOR (95% CI ) | AOR (95% CI ) | AOR (95% CI ) | AOR (95% CI ) |
| **Gender** | | | | | | | | | | | | | | |
| Male® | 1 | 1 | 1 | 1 | 1 | 1 | 1 | 1 | 1 | 1 | 1 | 1 | 1 | 1 |
| Female | 0.38 (0.07,1.99) | 0.53 (0.04,6.33) | 0.42**(0.18,0.98) | 2.27*(1.12,4.6) | 1.7 (0.9,3.22) | 0.62**(0.46,0.85) | 0.83 (0.47,1.46) | 0.91 (0.48,1.72) | 0.37*(0.17,0.81) | NA | 0.84 (0.14,5.09) | NA | 0.43 (0.16,1.13) | [46.48]***(5.77,374.2) |
| **Age** | | | | | | | | | | | | | | |
| 15-24® | 1 | 1 | 1 | 1 | 1 | 1 | 1 | 1 | 1 | 1 | 1 | 1 | 1 | 1 |
| 25-44 | 0.16 (0.02,1.12) | 0.03 (0,1.84) | 1.18 (0.46,3.05) | 0.83 (0.21,3.25) | 0.53 (0.24,1.17) | 0.31***(0.22,0.44) | 0.31***(0.17,0.57) | 0.42**(0.23,0.75) | 0.35**(0.17,0.72) | 2.23 (0.46,10.69) | 0.47 (0.07,3.18) | 0.51 (0.08,3.23) | 1.04 (0.5,2.14) | 1.28 (0.33,4.99) |
| 45-64 | 0.50 (0,78.42) | NA | 1.47 (0.51,4.24) | 1.16 (0.28,4.88) | 0.29*(0.11,0.74) | 0.12***(0.08,0.19) | 0.14***(0.06,0.3) | 0.34**(0.17,0.7) | 0.17*(0.04,0.67) | 4.54 (1,20.61) | 0.35 (0.04,2.87) | NA | 0.87 (0.34,2.23) | 2.27 (0.52,9.94) |
| above 64 | NA | NA | NA | NA | 0.05**(0.01,0.42) | 0.05***(0.02,0.13) | 0.17**(0.05,0.58) | 0.10**(0.02,0.5) | 0.04*(0,0.51) | NA | 2.36 (0.32,17.4) | NA | 1.4 (0.23,8.47) | NA |
| **Residence** | | | | | | | | | | | | | | |
| Urban® | 1 | 1 | 1 | 1 | 1 | 1 | 1 | 1 | 1 | 1 | 1 | 1 | 1 | 1 |
| Rural | 0.01**(0.00,0.15) | 4.66 (0.34,64.51) | NA | 1.35 (0.63,2.91) | 0.82 (0.44,1.55) | 0.59**(0.44,0.80) | 0.89 (0.53,1.51) | 0.55 (0.3,1) | 0.9 (0.36,2.23) | 0.55 (0.15,2.06) | 0.19 (0.03,1.21) | 0.25*(0.06,0.95) | 0.40**(0.21,0.78) | 4.37*(1.08,17.73) |
| **Education** | | | | | | | | | | | | | | |
| No formal education® | 1 | 1 | 1 | 1 | 1 | 1 | 1 | 1 | 1 | 1 | 1 | 1 | 1 | 1 |
| Completed primary | 0.16 (0.00,9.09) | [38.65](0.13,11919.09) | 0.21 (0.03,1.38) | 0.17 (0.02,1.65) | 0.76 (0.17,3.48) | 0.52*(0.28,0.98) | 0.1*(0.01,0.82) | 2.18 (0.29,16.55) | 0.13 (0.02,1.02) | 0.95 (0.23,3.92) | 0.21 (0.03,1.68) | 0.1*(0.01,0.89) | NA | 0.56 (0.08,4.04) |
| Completed secondary | 0.59 (0.00,113.42) | NA | 0.23 (0.04,1.29) | 0.44 (0.09,2.25) | 0.78 (0.2,3.08) | 0.68*(0.5,0.92) | 0.65 (0.36,1.15) | 2.94 (0.4,21.91) | 0.24 (0.04,1.38) | 0.58 (0.19,1.82) | 1.38 (0.12,15.99) | 0.06*(0,0.71) | 0.75 (0.41,1.38) | 0.33 (0.09,1.19) |
| Completed college/university | 0.41 (0.01,17.31) | [67.97](0.65,7054.79) | 0.24 (0.05,1.2) | 1.08 (0.21,5.52) | 1.28 (0.31,5.29) | NA | NA | 1.31 (0.16,10.51) | 0.39 (0.06,2.32) | NA | 1.21 (0.12,12.55) | 0.03*(0,0.73) | NA | NA |
| **Occupation** | | | | | | | | | | | | | | |
| Non employed® | 1 | 1 | 1 | 1 | 1 | 1 | 1 | 1 | 1 | 1 | 1 | 1 | 1 | 1 |
| Employed | 9.16 (0.29,284.89) | 16.73 (0.28,999.11) | 1.01 (0.49,2.09) | 1.8 (0.84,3.84) | 0.96 (0.41,2.26) | 1 (0.71,1.41) | 1.64 (0.94,2.88) | 0.69 (0.4,1.19) | 0.58 (0.28,1.2) | 0.41 (0.11,1.49) | 3.89 (0.44,34.75) | 6.65 (0.49,89.96) | 0.95 (0.43,2.08) | NA |
| **Wealth Index** | | | | | | | | | | | | | | |
| Rich® | 1 | 1 | 1 | 1 | 1 | 1 | 1 | 1 | 1 | 1 | 1 | 1 | 1 | 1 |
| Middle | 0.08(0,2.42) | NA | NA | NA | 0.68 (0.26,1.8) | 0.57*(0.35,0.94) | 1.46 (0.77,2.75) | 0.85 (0.37,1.94) | 1.02 (0.48,2.19) | 1.88 (0.47,7.57) | 1.29 (0.15,11.28) | 0.92 (0.27,3.17) | 0.52 (0.23,1.17) | 0.71 (0.17,3.02) |
| Poor | [58.64]***(6.99,491.99) | NA | NA | 0.73 (0.26,2.06) | 0.86 (0.41,1.81) | 0.95 (0.69,1.31) | 0.54 (0.24,1.21) | 0.62 (0.32,1.2) | 0.17**(0.05,0.54) | 0.47 (0.05,4.19) | 4.66 (0.28,76.22) | 0.29 (0.01,6.79) | 0.42 (0.16,1.08) | 0.76 (0.08,7.49) |
| **Currently smoke tobacco** | | | | | | | | | | | | | | |
| No® | 1 | 1 | 1 | 1 | 1 | 1 | 1 | 1 | 1 | 1 | 1 | 1 | 1 | 1 |
| Yes | 2.38 (0.08,68.27) | 2.93 (0.22,38.99) | [17.68]***(6.56,47.67) | 2.26*(1.01,5.04) | [16.97]***(7.46,38.59) | 5.60***(4.15,7.54) | 3.62***(2.04,6.41) | 6.66***(3.83,11.59) | 4.8***(2.36,9.76) | 5.33 (0.99,28.55) | 4.86*(1.12,21.14) | 13.42*(1.31,137.7) | 12.19***(5.27,28.24) | [458.5]***(27.08,7763.37) |
| **Advertisements in stores** | | | | | | | | | | | | | | |
| No® | 1 | 1 | 1 | 1 | 1 | 1 | 1 | 1 | 1 | 1 | 1 | 1 | 1 | 1 |
| Yes | NA | [17.29]*(1.5,198.96) | 1.45 (0.63,3.36) | 1.82 (0.92,3.61) | 0.99 (0.45,2.2) | 1.32 (0.83,2.09) | 1.71 (0.93,3.16) | 0.63 (0.23,1.67) | 1.18 (0.55,2.54) | 1.3 (0.55,3.07) | 0.97 (0.22,4.25) | 0.78 (0.17,3.65) | 0.86 (0.48,1.54) | 1.76 (0.4,7.74) |
| **Advertisements on internet** | | | | | | | | | | | | | | |
| No® | 1 | 1 | 1 | 1 | 1 | 1 | 1 | 1 | 1 | 1 | 1 | 1 | 1 | 1 |
| Yes | NA | NA | 1.07 (0.36,3.17) | 1.63 (0.44,6.13) | 0.63 (0.22,1.74) | 1.41 (0.96,2.07) | 0.61 (0.23,1.63) | 1.14 (0.31,4.28) | 1.18 (0.51,2.74) | NA | 0.72 (0.05,10.21) | 0.46 (0.05,3.88) | 2.96**(1.41,6.21) | 2.05 (0.1,41.01) |

***Note**: NA- data not available or cases not available; ® Reference Category; *** p<0.001, ** p<0.01, * p<0.05; [ ] AOR is typically high as the odds of e-cigarette use in the reference population is too low compared the predictor population and thus not interpretable in general.*



For peer review only - http://bmjopen.bmj.com/site/about/guidelines.xhtml

***Predicted average prevalence of e-cigarettes use in the countries***

From the country-specific logistic regression (Table 5), we predicted the average national prevalence for the respective countries. When adjusted for the socio-economic and demographic factors we found that Malaysia carried the highest burden of e-cigarette use (39/1000 population) compared to Indonesia (29/1000 population) during 2011. Country like the Russian Federation carried a prevalence of 37/1000 population during 2016 whereas in Ethiopia, the prevalence was 16/1000 population in the same year. Qatar from the Eastern Mediterranean region and Greece from the European region showed almost an equal prevalence of e-cigarette use during 2013. On the other hand, Kazakhstan during 2014 and Ukraine during 2017 carried a prevalence of 33 and 30 persons per thousand population of e-cigarette use (Table 5).

**Table 5.** Estimates of country level average prevalence of e-cigarette use and the total number of users, GATS, 2011-2017

| WHO Region | Country | Survey Year | Total Population* | Average prevalence (predicted) |
|---|---|---|---|---|
| African Region | Ethiopia | 2016 | 103,603,501 | 0.016 |
| | Senegal | 2017 | 15,419,381 | 0.007 |
| Eastern Mediterranean | Qatar | 2013 | 2,336,574 | 0.018 |
| European Region | Greece | 2013 | 10,965,211 | 0.019 |
| | Kazakhstan | 2014 | 17,288,285 | 0.033 |
| | Russian Federation | 2016 | 144,342,396 | 0.037 |
| | Ukraine | 2017 | 44,831,135 | 0.030 |
| Region of the Americas | Costa Rica | 2015 | 4,847,804 | 0.024 |
| | Mexico | 2015 | 124,777,324 | 0.017 |
| South-east Asia Region | Indonesia | 2011 | 245,116,206 | 0.029 |
| | India® | 2017 | 1,338,658,835 | 0.012 |
| Western Pacific Region | Malaysia | 2011 | 28,650,955 | 0.039 |
| | Philippines | 2015 | 102,113,212 | 0.022 |
| | Vietnam | 2015 | 92,677,076 | 0.012 |

***Note:** ®: Reference Country;*
* https://data.worldbank.org/indicator/SP.POP.TOTL?most_recent_year_desc=false;

## Discussion

This study has examined the awareness of e-cigarette use, prevalence, and socio-demographic determinants utilizing the most recent round of the GATS surveys covering 14 selected countries. The developed nations like USA, UK, New Zealand, France, and Germany have their own individual surveys to provide general/population-specific estimates of e-cigarette use and awareness. It was estimated that the prevalence of e-cigarette use among USA middle and high school students increased from 3% in 2011 to 7% in 2012 [41]. According to the Local Tobacco Control Profiles for England annual report, there had been a significant increase in e-cigarette users (population aged ≥16) from 15% in 2014 to 19% in 2017 in UK. Additionally, it had also been found that use of e-cigarette was much more prevalent among the younger population (aged 16-24 years) than the older



For peer review only - http://bmjopen.bmj.com/site/about/guidelines.xhtml

population (aged ≥ 60 years) [42]. Quatremere et al., 2019 estimated that 4% of the population in Mainland France were e-cigarette user and 3% of them used it on a daily basis. Of these total users, 60% were men, and 58% had a bachelor's degree [43]. Eichler et al., 2016 using a population-based cross-sectional survey, conducted during 2016, found that 49% of males and 51% of females used e-cigarettes in Germany [44]. In New Zealand, 7% of the general population used e-cigarettes, with young persons (18-24 years) were more likely than those over 45 years [45]. Furthermore, independent of educational qualification, persons with higher income were more likely to use e-cigarettes than those with a moderate or lower income in New Zealand [45].

This study suggests that countries in the European and eastern Mediterranean regions have a higher awareness of e-cigarette than the countries of other regions. The awareness and current use of e-cigarettes are higher among urban population in almost all the countries studied. On the contrary, Greece and Vietnam show higher prevalence of awareness in the rural areas. It is possible that the exposure to advertisement, accessibility and availability of e-cigarettes may have led to higher e-cigarettes use in urban areas [46].

The countries of European region demonstrate higher prevalence of e-cigarette use with higher education level in the population. In addition, the wealthy population are more likely to use e-cigarettes. The prevalence of current e-cigarette use may be higher in Russian Federation (4.39%) because e-cigarettes are not covered under the tobacco control policy of Federal Law No. 15-FZ [47]. Therefore, the restriction on marketing and advertisement finds a policy gap [33]. Though e-cigarette regulation policies in Malaysia (3.94%) and in Ukraine (3.28%), puts a strict restrictions on distribution, importation, minimum age and in sales; yet the prevalence of e-cigarette use is moderately high in these two countries [48]. On the contrary, the least prevalence of e-cigarettes use in the countries like India (0.66%) and Vietnam (1.88%) is may be due to the ban on manufacturing, imports, sales, advertisement, and in distribution [48–50].

The statistical evidence from this study suggest that the urban population, males, young people (15-24), those with higher wealth scores and the higher educated individuals keep better knowledge of e-cigarettes across all the selected countries. Males have higher prevalence of e-cigarette use than the females, except in Greece. A study by Tzortzi et al., 2020 reported that both tobacco smoking and e-cigarette use among females are higher than males in Greece, which is attributable to better economic condition [51]. A study based on an online survey in USA, had found that male older adults use e-cigarettes as a medium to quit smoking while the initiation of e-cigarette use among females is influenced by their family and friends [52]. The present study has shown that e-cigarette use among the younger adults is high compared to the older adults across all the selected countries; which emphasizes the need of implementing new population specific policies to curb the use of nicotine at younger ages. Notably, the cross-country analysis does not show consistent association between use of e-cigarette and educational qualification across selected GATS countries. While, the wealthy population are more likely to use e-cigarettes.

The multivariate adjusted model also reveals the association of e-cigarettes with gender, age, resident type, and employment. Results also indicate that males have higher use of e-cigarette which could be due to quitting



For peer review only - http://bmjopen.bmj.com/site/about/guidelines.xhtml

tobacco smoking. In the counterpart, women's initiation of e-cigarette use is often restricted by social stigma and health concerns [53]. On the other hand, males purchase e-cigarettes independently while females rely on their peers, limiting their use [54,55]. In Costa Rica and Mexico, young adults use e-cigarettes, suggesting a relatively higher availability, affordability and moderate restrictive policies on nicotine vaping products [56]. Previous studies on tobacco smoking suggest that urban youths are more likely to use tobacco than their counterparts which may be due to urban accessibility, urban space, and advertisements [34,57]. Literatures also suggests that early employment is associated with daily use among youths [58]. Adolescents/youths who are either employed or receive more pocket money and supposedly enjoy economic independence are more likely to smoke and use other addictive substances [58,59]. The present study shows that the unemployed population has a lower prevalence of e-cigarette use, possibly due to a lack of discretionary income [60,61]. The findings are also in line with other studies explaining the association of the advertisement/promotions through various media that trigger the use of e-cigarette among young adults [54,62,63]. The use of the internet and social media for advertising, marketing, and ENDS promotion has expanded rapidly and concerns have been raised regarding deceptive health statements, claims on withdrawal effectiveness, and hence targeting the youths [64]. Although the ill-health effects of e-cigarette use are debated, it is still established that the aerosols of the majority of ENDS contain toxic chemicals that are hazardous to lung health and elevate the risk of cardiovascular diseases [65,66]. The promotion and marketing challenges of e-cigarettes revolve around the country's public experiences and tobacco control measures. In many countries, such as Australia, Brazil, Denmark, and others, e-cigarettes advertising and marketing are illegal [48]. On the other hand, sales in other countries are legal and authorized [67]. Interestingly, in United Kingdom (UK), despite a strong tobacco policy, e-cigarettes use is booming [14]. At the same time, countries like Costa Rica, Ecuador, Honduras, the Republic of Korea, Togo and Vietnam do not have regulations on sales of e-cigarettes [48,67]. Initially, the e-cigarettes manufacturers were independent and then transgressional tobacco companies came to venture into the market by creating a new form of nicotine consumers (young and non-smokers) pretending to curtail the smoking epidemic as a reputational tactic [68]. Although the present study is cross-sectional, it fails to capture the association of purchasing behavior and employment. Therefore, it generalizes the likelihood of economic stability and e-cigarette use across selected GATS countries.

An important strength of this study is that it provides important baseline e-cigarette prevalence data for multiple countries. As GATS data collections continue, the prevalence of e-cigarette use in each country can continue to be monitored and can be used to evaluate policy changes that occur. However, this study has some limitations. Firstly, we have considered only 14 countries in the temporal setting of 2011-2017. Thereafter, we could not incorporate the drastic changes in e-cigarette devices, brands, marketing between 2011-2017, which might have impacted the prevalence of e-cigarette use. Lastly, the outcome variables in this particular study are self-reported in nature, thus the study estimates are limited to self-reporting bias and system bias. The self-reporting bias among the individuals largely depends on their socio-economic and demographic characteristics, which has been controlled in the study. To address the system bias in the estimations, we utilized the country-specific survey

For peer review only

weights to derive the estimates. Notably, the GATS surveys are popularly referred and used across countries to provide the prevalence of tobacco and e-cigarette use in the surveyed population utilizing the self-reported information only. At the same time, according to the WHO-GATS survey protocol, the survey instruments were developed to collect the necessary information on e-cigarette awareness and its use and thus we are restricted to utilize these self-reported information in this study in the absence of any other relevant data for these countries. Nevertheless, despite a few lacunae, there are gaps in the knowledge of cross-country e-cigarette use and this study provides useful clues to policymakers for establishing effective measures for public health planning.

## Conclusion

The findings from this study show that e-cigarette use is widespread across these selected GATS countries, with a higher prevalence among young adults. At the same time, while exploring the country-specific patterns in the determinants of e-cigarette use; economic wellbeing and higher educational attainment have emerged as consistent predictors of e-cigarette use. With a cross-country analysis of GATS datasets, this study presents the most recent socio-economic and demographic patterns of e-cigarettes use and its determinants. In addition, this study identifies the sub-populations at higher risk of e-cigarette use so that policymakers and health professionals can be more assertive in their interventions. As e-cigarette use is much more prevalent among young adults, a community level surveillance is needed to reduce the burden of e-cigarette use among males. Also, an apt awareness intervention should be designed to ensure no further uptake of e-cigarette use among non-smokers.

## Declarations

### Ethics approval and consent to participate

*Not applicable.*

### Consent for publication

*Not applicable.*

### Availability of data and materials

*The datasets used and/or analysed during the current study are available from the corresponding author on reasonable request.*

### Competing interests

*The authors declare that they have no competing interests.*

### Funding

*This research received no specific grant from any funding agency in the public, commercial, or not-for-profit sectors.*



For peer review only - http://bmjopen.bmj.com/site/about/guidelines.xhtml

**Author contributions**

SK, SS, and JK, AC and EAB: Conceptualization, methodology, validation, formal analysis, investigation, writing (original draft preparation). AC: Review and editing. EAB: Conceptualization, methodology, validation, formal analysis and writing (review and editing). BP: Review. All authors have read and approved the final manuscript.

**Acknowledgements**

The authors would like to thank and acknowledge International Institute for Population Sciences , Mumbai, India, for funding the study for publication.

For peer review only

## References


1 Caraballo RS, Jamal A, Nguyen KH, *et al.* Electronic Nicotine Delivery System Use Among U.S. Adults, 2014. *Am J Prev Med* 2016;**50**:226–9. doi:10.1016/j.amepre.2015.09.013

2 Singh T, Arrazola RA, Corey CG, *et al.* Tobacco Use Among Middle and High School Students — United States, 2011–2015. 2016;**65**:8.

3 Morean M, Butler E, Bold K, *et al.* Preferring more e-cigarette flavors is associated with e-cigarette use frequency among adolescents but not adults. 2018;:10.

4 Hartwell G, Thomas S, Egan M, *et al.* E-cigarettes and equity: a systematic review of differences in awareness and use between sociodemographic groups. *Tob Control* 2017;**26**:e85–91. doi:10.1136/tobaccocontrol-2016-053222

5 U.S. Department of Health and Human Services. E-Cigarette Use Among Youth and Young Adults. A Report of the Surgeon General. Cent. Dis. Control Prev. 2017.https://www.cdc.gov/tobacco/data_statistics/sgr/e-cigarettes/index.htm (accessed 26 May 2021).

6 Mirbolouk M, Charkhchi P, Kianoush S, *et al.* Prevalence and Distribution of E-Cigarette Use Among U.S. Adults: Behavioral Risk Factor Surveillance System, 2016. *Ann Intern Med* 2018;**169**:429–38. doi:10.7326/M17-3440

7 McMillen RC, Gottlieb MA, Winickoff JP. e-Cigarettes—The Roles of Regulation and Clinicians. *JAMA Intern Med* 2015;**175**:1603–4. doi:10.1001/jamainternmed.2015.4436

8 Liu X, Lugo A, Davoli E, *et al.* Electronic cigarettes in Italy: a tool for harm reduction or a gateway to smoking tobacco? *Tob Control* 2020;**29**:148–52. doi:10.1136/tobaccocontrol-2018-054726

9 Bhatnagar A, Payne TJ, Robertson RM. Is There A Role for Electronic Cigarettes in Tobacco Cessation? *J Am Heart Assoc* 2019;**8**:e012742. doi:10.1161/JAHA.119.012742

10 Camenga DR, Kong G, Cavallo DA, *et al.* Current and Former Smokers' Use of Electronic Cigarettes for Quitting Smoking: An Exploratory Study of Adolescents and Young Adults. *Nicotine Tob Res Off J Soc Res Nicotine Tob* 2017;**19**:1531–5. doi:10.1093/ntr/ntw248

11 Hartmann-Boyce J, Begh R, Aveyard P. Electronic cigarettes for smoking cessation. *BMJ* 2018;**360**:j5543. doi:10.1136/bmj.j5543

12 Mayer M, Reyes-Guzman C, Grana R, *et al.* Demographic Characteristics, Cigarette Smoking, and e-Cigarette Use Among US Adults. *JAMA Netw Open* 2020;**3**:e2020694. doi:10.1001/jamanetworkopen.2020.20694

13 Etter J-F. Electronic cigarettes: a survey of users. *BMC Public Health* 2010;**10**:231. doi:10.1186/1471-2458-10-231

14 WHO Framework Convention on Tobacco Control, Conference of the Parties to the WHO Framework Convention on Tobacco Control, sixth session. (2014). Electronic nicotine delivery systems: Report by WHO. Published Online First: 2014.https://apps.who.int/iris/handle/10665/147110 (accessed 26 May 2021).

15 National Academies of Sciences, Engineering, and Medicine, Health and Medicine Division, Board on Population Health and Public Health Practice, *et al. Public Health Consequences of E-Cigarettes*. Washington (DC): : National Academies Press (US) 2018. http://www.ncbi.nlm.nih.gov/books/NBK507171/ (accessed 26 May 2021).




For peer review only - http://bmjopen.bmj.com/site/about/guidelines.xhtml

16 Suter MA, Mastrobattista J, Sachs M, *et al.* Is There Evidence for Potential Harm of Electronic Cigarette Use in Pregnancy? *Birt Defects Res A Clin Mol Teratol* 2015;**103**:186–95. doi:10.1002/bdra.23333

17 Pepper JK, Ribisl KM, Brewer NT. Adolescents' interest in trying flavoured e-cigarettes. *Tob Control* 2016;**25**:ii62–6. doi:10.1136/tobaccocontrol-2016-053174

18 Jones K, Salzman GA. The Vaping Epidemic in Adolescents. *Mo Med* 2020;**117**:56–8.https://www.ncbi.nlm.nih.gov/pmc/articles/PMC7023954/

19 Berry KM, Fetterman JL, Benjamin EJ, *et al.* Association of Electronic Cigarette Use With Subsequent Initiation of Tobacco Cigarettes in US Youths. *JAMA Netw Open* 2019;**2**:e187794. doi:10.1001/jamanetworkopen.2018.7794

20 Gentzke AS. Vital Signs: Tobacco Product Use Among Middle and High School Students — United States, 2011–2018. *MMWR Morb Mortal Wkly Rep* 2019;**68**. doi:10.15585/mmwr.mm6806e1

21 Simon P, Camenga DR, Morean ME, *et al.* Socioeconomic status and adolescent e-cigarette use: The mediating role of e-cigarette advertisement exposure. *Prev Med* 2018;**112**:193–8. doi:10.1016/j.ypmed.2018.04.019

22 Kock L, Shahab L, West R, *et al.* E-cigarette use in England 2014–17 as a function of socio-economic profile. *Addiction* 2019;**114**:294–303. doi:https://doi.org/10.1111/add.14446

23 Rogers EM, Singhal A, Quinlan MM. *Diffusion of Innovations*. Routledge 2014. doi:10.4324/9780203887011-36

24 Thun M, Peto R, Boreham J, *et al.* Stages of the cigarette epidemic on entering its second century. *Tob Control* 2012;**21**:96–101. doi:10.1136/tobaccocontrol-2011-050294

25 Pepper JK, Emery SL, Ribisl KM, *et al.* Effects of advertisements on smokers' interest in trying e-cigarettes: the roles of product comparison and visual cues. 2014;:6.

26 Duke JC, Lee YO, Kim AE, *et al.* Exposure to Electronic Cigarette Television Advertisements Among Youth and Young Adults. *PEDIATRICS* 2014;**134**:e29–36. doi:10.1542/peds.2014-0269

27 Farrelly MC, Duke JC, Crankshaw EC, *et al.* A Randomized Trial of the Effect of E-cigarette TV Advertisements on Intentions to Use E-cigarettes. *Am J Prev Med* 2015;**49**:686–93. doi:10.1016/j.amepre.2015.05.010

28 Robertson L, Cameron C, McGee R, *et al.* Point-of-sale tobacco promotion and youth smoking: a meta-analysis. *Tob Control* 2016;**25**:e83–9. doi:10.1136/tobaccocontrol-2015-052586

29 WHO report on the global tobacco epidemic, 2015: Rising Taxes on Tobacco. WHO. 2015.http://www.who.int/tobacco/global_report/2015/en/ (accessed 26 May 2021).

30 WHO report on the global tobacco epidemic, 2017: Monitoring tobacco use and prevention policies. WHO. 2017.http://www.who.int/tobacco/global_report/2017/en/ (accessed 26 May 2021).

31 Report of the seventh session of the Conference of the Parties to the WHO Framework Convention on Tobacco Control Delhi, India, 7–12 November 2016. WHO. 2016.http://www.who.int/fctc/cop/sessions/cop7/cop7-summary/en/ (accessed 26 May 2021).

32 WHO report on the global tobacco epidemic, 2019: Offer help to quit tobacco use. 2019.https://www.who.int/teams/health-product-and-policy-standards/access-to-assistive-technology-medical-devices/health-promotion (accessed 26 May 2021).

For peer review only



For peer review only - http://bmjopen.bmj.com/site/about/guidelines.xhtml

33 Lagasse LP, Grant AS, Minosa MKR, *et al.* Availability and advertising of electronic cigarettes in two Russian cities following implementation of comprehensive tobacco advertising restrictions. *Tob Induc Dis* 2020;**18**:4. doi:10.18332/tid/115794

34 Noland M, Rayens MK, Wiggins AT, *et al.* Current Use of E-Cigarettes and Conventional Cigarettes Among US High School Students in Urban and Rural Locations: 2014 National Youth Tobacco Survey. *Am J Health Promot* 2018;**32**:1239–47. doi:10.1177/0890117117719621

35 Palipudi KM, Mbulo L, Morton J, *et al.* Awareness and Current Use of Electronic Cigarettes in Indonesia, Malaysia, Qatar, and Greece: Findings From 2011–2013 Global Adult Tobacco Surveys. *Nicotine Tob Res* 2016;**18**:501–7. doi:10.1093/ntr/ntv081

36 King BA, Mirza SA, Babb SD, *et al.* A cross-country comparison of secondhand smoke exposure among adults: findings from the Global Adult Tobacco Survey (GATS). *Tob Control* 2013;**22**:e5–e5. doi:10.1136/tobaccocontrol-2012-050582

37 Kostova D, Chaloupka FJ, Yurekli A, *et al.* A cross-country study of cigarette prices and affordability: evidence from the Global Adult Tobacco Survey. *Tob Control* 2014;**23**:e3–e3. doi:10.1136/tobaccocontrol-2011-050413

38 Morton J, Song Y, Fouad H, *et al.* Cross-country comparison of waterpipe use: nationally representative data from 13 low and middle-income countries from the Global Adult Tobacco Survey (GATS). *Tob Control* 2014;**23**:419–27. doi:10.1136/tobaccocontrol-2012-050841

39 Rutstein SO, Johnson K. The DHS wealth index. Published Online First: 1 August 2004.https://dhsprogram.com/publications/publication-cr6-comparative-reports.cfm (accessed 2 Sep 2021).

40 Rutstein SO. Steps to constructing the new DHS Wealth Index. 2015.https://dhsprogram.com/programming/wealth%20index/Steps_to_constructing_the_new_DHS_Wealth_Index.pdf (accessed 2 Sep 2021).

41 Corey C, Wang B, Johnson SE, *et al.* Notes from the Field: Electronic cigarette use among middle and high school students - United States, 2011-2012. *Morb Mortal Wkly Rep* 2013;**62**:729–30.https://jhu.pure.elsevier.com/en/publications/notes-from-the-field-electronic-cigarette-use-among-middle-and-hi-5 (accessed 1 Sep 2021).

42 Cornish D, Brookman A, Horton M, *et al.* Adult smoking habits in the UK: 2017. Office For National Statistics: Office For National Statistics. 2018.https://www.ons.gov.uk/peoplepopulationandcommunity/healthandsocialcare/drugusealcoholandsmoking/datasets/ecigaretteuseingreatbritain (accessed 31 Aug 2021).

43 Quatremère G, Pasquereau A, Guignard R, *et al.* Use and perceptions of electronic cigarette by the adult population in France in 2017. *Eur J Public Health* 2019;**29**. doi:10.1093/eurpub/ckz186.626

44 Eichler M, Blettner M, Singer S. The Use of E-Cigarettes. *Dtsch Ärztebl Int* 2016;**113**:847–54. doi:10.3238/arztebl.2016.0847

45 Li J, Bullen C, Newcombe R, *et al.* The use and acceptability of electronic cigarettes among New Zealand smokers. *N Z Med J* 2013;**126**:48–57.

46 Collins L, Glasser AM, Abudayyeh H, *et al.* E-Cigarette Marketing and Communication: How E-Cigarette Companies Market E-Cigarettes and the Public Engages with E-cigarette Information. *Nicotine Tob Res* 2019;**21**:14–24. doi:10.1093/ntr/ntx284



For peer review only - http://bmjopen.bmj.com/site/about/guidelines.xhtml

47 Russian Federation - Federal Law No.15-FZ of 23 February 2013 on Health Protection from Exposure to Environmental Tobacco Smoke and the Consequences of Tobacco Consumption. 2013.http://www.ilo.org/dyn/natlex/natlex4.detail?p_lang=en&p_isn=96223&p_country=RUS&p_count=1 (accessed 27 May 2021).

48 Kennedy RD, Awopegba A, León ED, *et al.* Global approaches to regulating electronic cigarettes. *Tob Control* 2017;**26**:440–5. doi:10.1136/tobaccocontrol-2016-053179

49 Dyer O. India bans e-cigarettes by executive order. *BMJ* 2019;**366**:l5649. doi:10.1136/bmj.l5649

50 Jha P. Smoking cessation and e-cigarettes in China and India. *BMJ* 2019;**367**:l6016. doi:10.1136/bmj.l6016

51 Tzortzi A, Kapetanstratak M, Evangelopoulou V, *et al.* Smoking in Greece Where we stand in 2020. 2020;**33**:9.http://www.pneumon.org/arpil-june-2020-vol-33-issue-2/newsid789/790

52 Piñeiro B, Correa JB, Simmons VN, *et al.* Gender differences in use and expectancies of e-cigarettes: Online survey results. *Addict Behav* 2016;**52**:91–7. doi:10.1016/j.addbeh.2015.09.006

53 Bauhoff S, Montero A, Scharf D. Perceptions of e-cigarettes: a comparison of adult smokers and non-smokers in a Mechanical Turk sample. *Am J Drug Alcohol Abuse* 2017;**43**:311–23. doi:10.1080/00952990.2016.1207654

54 Kong G, Morean ME, Cavallo DA, *et al.* Sources of Electronic Cigarette Acquisition among Adolescents in Connecticut. *Tob Regul Sci* 2017;**3**:10–6. doi:10.18001/TRS.3.1.2

55 Kong G, Kuguru KE, Krishnan-Sarin S. Gender Differences in U.S. Adolescent E-Cigarette Use. *Curr Addict Rep* 2017;**4**:422–30. doi:10.1007/s40429-017-0176-5

56 Gravely S, Driezen P, Ouimet J, *et al.* Prevalence of awareness, ever-use and current use of nicotine vaping products (NVPs) among adult current smokers and ex-smokers in 14 countries with differing regulations on sales and marketing of NVPs: cross-sectional findings from the ITC Project. *Addiction* 2019;**114**:1060–73. doi:https://doi.org/10.1111/add.14558

57 Pesko MF, Robarts AMT. Adolescent Tobacco Use in Urban Versus Rural Areas of the United States: The Influence of Tobacco Control Policy Environments. *J Adolesc Health* 2017;**61**:70–6. doi:10.1016/j.jadohealth.2017.01.019

58 Wu L, Schlenger W, Galvin D. The relationship between employment and substance use among students aged 12 to 17*1. *J Adolesc Health* 2003;**32**:5–15. doi:10.1016/S1054-139X(02)00447-0

59 Scragg R, Laugesen M, Robinson E. Cigarette smoking, pocket money and socioeconomic status: results from a national survey of 4th form students in 2000. 2002;**115**:8.

60 Griesbach D, Amos A, Currie C. Adolescent smoking and family structure in Europe. *Soc Sci Med* 2003;**56**:41–52. doi:10.1016/S0277-9536(02)00014-X

61 Darling H, Reeder AI, McGee R, *et al.* Brief report: Disposable income, and spending on fast food, alcohol, cigarettes, and gambling by New Zealand secondary school students. *J Adolesc* 2006;**29**:837–43. doi:10.1016/j.adolescence.2006.06.003

62 Ashford K, Rayens E, Wiggins AT, *et al.* Advertising exposure and use of e-cigarettes among female current and former tobacco users of childbearing age. *Public Health Nurs* 2017;**34**:430–6. doi:https://doi.org/10.1111/phn.12334



For peer review only - http://bmjopen.bmj.com/site/about/guidelines.xhtml

63 Dai H, Hao J. Exposure to Advertisements and Susceptibility to Electronic Cigarette Use Among Youth. *J Adolesc Health* 2016;**59**:620–6. doi:10.1016/j.jadohealth.2016.06.013

64 WHO. E-cigarettes are harmful to health. 2020.https://www.who.int/news/item/05-02-2020-e-cigarettes-are-harmful-to-health (accessed 27 May 2021).

65 MacDonald A, Middlekauff HR. Electronic cigarettes and cardiovascular health: what do we know so far? *Vasc Health Risk Manag* 2019;**15**:159–74. doi:10.2147/VHRM.S175970

66 Buchanan ND, Grimmer JA, Tanwar V, *et al.* Cardiovascular risk of electronic cigarettes: a review of preclinical and clinical studies. *Cardiovasc Res* 2020;**116**:40–50. doi:10.1093/cvr/cvz256

67 Institute for Global Tobacco Control. Country Laws Regulating E-cigarettes: A Policy Scan. Baltimore, MD: Johns Hopkins Bloomberg School of Public Health. Glob. Tob. Control - Learn. Experts. 2015.https://www.globaltobaccocontrol.org/e-cigarette_policyscan (accessed 26 May 2021).

68 Peeters S, Gilmore AB. Understanding the emergence of the tobacco industry's use of the term tobacco harm reduction in order to inform public health policy. *Tob Control* 2015;**24**:182–9. doi:10.1136/tobaccocontrol-2013-051502

For peer review only



For peer review only - http://bmjopen.bmj.com/site/about/guidelines.xhtml

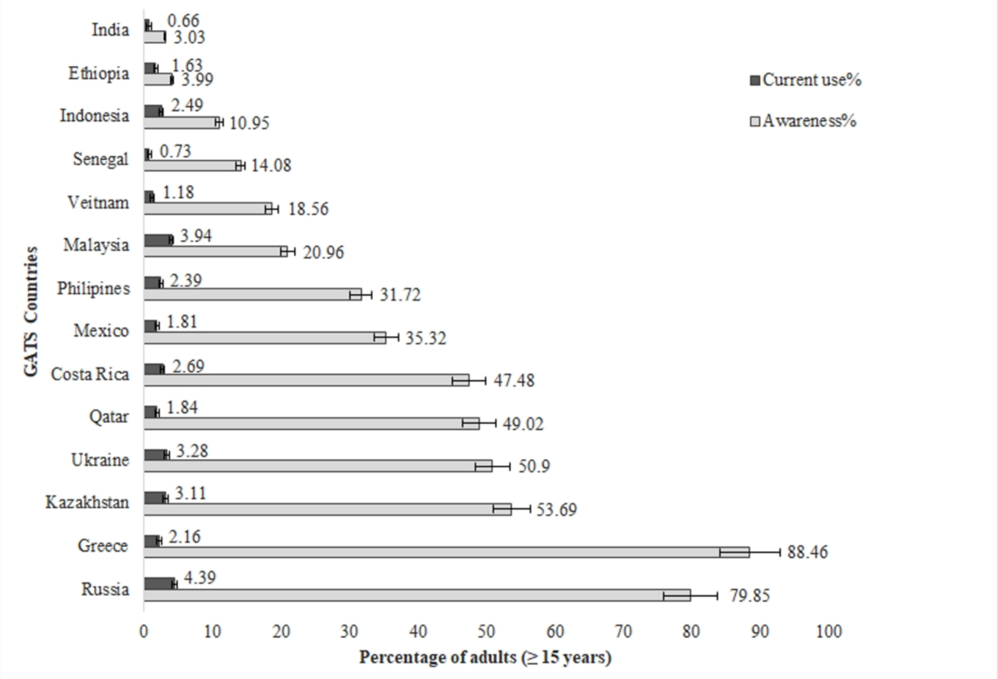


The prevalence of e-cigarettes usage and awareness across selected GATS countries

525x358mm (236 x 236 DPI)
